# Performance Evaluation of Host Biomarker Combinations for the Diagnosis of Serious Bacterial Infection in Young Febrile Children: A Double-Blind, Multicentre, Observational Study

**DOI:** 10.3390/jcm11216563

**Published:** 2022-11-04

**Authors:** Aurélie Portefaix, Sylvie Pons, Antoine Ouziel, Romain Basmaci, Philippe Rebaud, Marie-Caroline Delafay, Laurence Generenaz, Guy Oriol, Boris Meunier, Fatima Abbas-Chorfa, Sophie Trouillet-Assant, Tiphanie Ginhoux, Fabien Subtil, Yves Gillet, Karen Brengel-Pesce, Etienne Javouhey

**Affiliations:** 1 Clinical Investigation Center (CIC 1407), Hospices Civils de Lyon, 69677 Bron, France; 2 Laboratoire de Biométrie et Biologie Evolutive, CNRS, UMR 5558, Claude Bernard University of Lyon, 69622 Villeurbanne, France; 3 Joint Research Unit Hospices Civils de Lyon—bioMérieux, Hôpital Lyon Sud, 69795 Pierre-Bénite, France; 4 Joint Research Unit HCL-bioMérieux, EA 7426 “Pathophysiology of Injury-Induced Immunosuppression”, Hospices Civils de Lyon—bioMérieux, Université Claude Bernard Lyon 1, 69003 Lyon, France; 5 Pediatric Emergency and Intensive Care Unit, Hôpital Femme Mère Enfant, Hospices Civils of Lyon, 69677 Bron, France; 6 Service de Pédiatrie-Urgences, Hôpital Louis-Mourier—Assistance Publique Hôpitaux de Paris (APHP), 92700 Colombes, France; 7 Infections Antimicrobials Modelling Evolution (IAME), INSERM, UMR1137, Université de Paris, 75006 Paris, France; 8 Service de Pédiatrie-Néonatologie, Hôpital Nord-Ouest, Plateau d’Ouilly Gleizé BP 436, 69655 Villefranche-sur-Saône, France; 9 Service de Biostatistique, Hospices Civils de Lyon, 69003 Lyon, France; 10 Centre International de Recherche en Infectiologie (CIRI), INSERM U1111, CNRS UMR5308, ENS Lyon, Université Claude Bernard Lyon 1, 69364 Lyon, France

**Keywords:** paediatric serious bacterial infection, bacterial- and viral-induced host biomarkers, machine learning, C-reactive protein (CRP), procalcitonin (PCT), interleukin 6 (IL-6), neutrophil gelatinase-associated lipocalin-2 (NGAL), myxovirus resistance protein 1 (MxA), tumor necrosis factor-related apoptosis-inducing ligand (TRAIL), IFN-γ-induced protein 10 (IP-10)

## Abstract

The diagnosis of serious bacterial infection (SBI) in young febrile children remains challenging. This prospective, multicentre, observational study aimed to identify new protein marker combinations that can differentiate a bacterial infection from a viral infection in 983 children, aged 7 days–36 months, presenting with a suspected SBI at three French paediatric emergency departments. The blood levels of seven protein markers (CRP, PCT, IL-6, NGAL, MxA, TRAIL, IP-10) were measured at enrolment. The patients received the standard of care, blinded to the biomarker results. An independent adjudication committee assigned a bacterial vs. viral infection diagnosis based on clinical data, blinded to the biomarker results. Computational modelling was applied to the blood levels of the biomarkers using independent training and validation cohorts. Model performances (area under the curve (AUC), positive and negative likelihood ratios (LR+ and LR–)) were calculated and compared to those of the routine biomarkers CRP and PCT. The targeted performance for added value over CRP or PCT was LR+ ≥ 5.67 and LR− ≤ 0.5. Out of 652 analysed patients, several marker combinations outperformed CRP and PCT, although none achieved the targeted performance criteria in the 7 days–36 months population. The models seemed to perform better in younger (7–91 day-old) patients, with the CRP/MxA/TRAIL combination performing best (AUC 0.895, LR+ 10.46, LR− 0.16). Although computational modelling using combinations of bacterial- and viral-induced host-protein markers is promising, further optimisation is necessary to improve SBI diagnosis in young febrile children.

## 1. Introduction

Fever is one of the leading causes of consultation in paediatric emergency departments (PED) [1]. In febrile infants younger than 3 months of age, the prevalence of serious bacterial infection (SBI) ranges from 7% to 28% [1,2,3]. The differential diagnosis of SBI vs. benign viral infections in young febrile children remains challenging. Clinical prediction and risk stratification tools have been developed to guide antibiotherapy decision-making. These tools include medical history, clinical examination, and laboratory data, either evaluated alone (e.g., Rochester or Philadelphia criteria) [1,4] or in combination with the blood levels of biomarkers of bacterial infection such as C-reactive protein (CRP) and procalcitonin (PCT) (e.g., Lab-score, Step-by-Step approach) [1,3,5,6,7]. The diagnostic value of CRP and PCT as single markers has also been extensively evaluated [1,2,8]. In one of the largest prospective studies, including 7–91 day-old febrile children (*n* = 2047), CRP and PCT alone reached a sensitivity and specificity ranging from 74% to 78%, with positive and negative likelihood ratios (LR+, LR–) of 3.1–3.3 and 0.3, respectively [2]. While single markers of bacterial infection cannot reliably diagnose or exclude SBI, combined approaches have shown some value, notably in identifying patients with a low-risk of SBI or in ruling out SBI [1,5,8,9,10]. However, none of these approaches have reached an acceptable diagnostic accuracy for either the sensitivity or specificity needed for the identification of SBI in young febrile children [1,11]. Misdiagnosis of SBI is a major health burden, as under-treatment is associated with increased morbidity and mortality, while over-treatment is associated with antibiotic resistance, microbiota imbalance, and toxicity in young children [12,13,14,15].

Alternative diagnostic approaches have been investigated. Among them, host RNA biomarkers demonstrated their potential diagnostic value [1,16,17,18,19,20]. Although preliminary investigations are promising, the implementation of transcriptional signature-based assays in clinical routine using conventional RT-PCR might be limited by their practicability and extended turnaround time [1,20]. More recently, machine learning-based algorithms have been developed using multiple features, integrating either clinical parameters with biomarkers or combinations of protein biomarkers [11,16,21,22,23,24,25,26]. Notably, models combining the markers of host immune response to bacterial and viral infections demonstrated superior performance over routine biomarkers, to differentiate bacterial from viral infection among young febrile children [11,22,25,26]. Hence, the triple host-protein ImmunoXpert assay, combining the virally induced proteins tumor necrosis factor-related apoptosis-inducing ligand (TRAIL) and IFN-γ-induced protein 10 (IP-10) with the bacterially induced protein CRP, showed around 90% sensitivity and specificity in diagnosing a bacterial infection from a viral infection in paediatric patients [11,22,25,27,28,29]. Similarly, the point-of-care assay FebriDx, combining the bacterially induced protein CRP and the virally induced protein myxovirus resistance protein 1 (MxA), showed good performance in both children and adults with upper respiratory infections [11,26]. Although large cohort validation studies are still needed, these models support a new paradigm for the identification of diagnostic tools with improved accuracy, using machine learning computational modelling to integrate the host-based response markers of both bacterial and viral infections.

The goal of this study was to identify new marker combinations that can differentiate a bacterial infection from a viral infection in children younger than 3 years of age who are suspected of SBI, with a performance superior to that of the routine bacterial-induced markers CRP and/or PCT that are measured during a clinical examination. We hypothesised that computational modelling applied to bacteria-induced (CRP, PCT, interleukin 6 (IL-6), the urinary tract infection marker neutrophil gelatinase-associated lipocalin-2 (NGAL—also known as Lipocalin-2 or HNL)) [1,2,8,16,30,31,32,33], and virus-induced (MxA, TRAIL, IP-10) [16,22,24,26] markers selected based on their reported performance could improve the diagnosis of SBI. The performance of the validated models to diagnose bacterial vs. viral infections, as adjudicated by an expert panel, was compared to that of CRP and PCT single markers.

## 2. Materials and Methods

### 2.1. Study Design and Participants

A prospective, multicentre, cohort study was conducted in three French PED (Hôpital Femme Mère Enfant, Hospices Civils de Lyon, Bron; Hôpital Louis Mourier, Colombes; Hôpital Nord-Ouest, Villefranche-sur-Saône) between 6 June 2017 and 12 June 2019 (clinicaltrials.gov ID: NCT03163628). Children with national health insurance coverage, aged 7 days to 36 months, presenting to PED with suspected SBI and prescribed blood withdrawal as part of standard care were enrolled. Suspected SBI was defined as fever > 38°C for more than 6 h in children aged 7 days to 3 months and fever ≥38.5°C for more than 6 h but less than 7 days in children aged 3 to 36 months. Exclusion criteria included antibiotics administration within the past 48 h, vaccination with an inactivated vaccine within the past 48 h or with the MMR vaccine within the past 10 days, chronic inflammatory or immune disease (immunodeficiency, auto-immune disease), and surgery in the past 7 days. Written informed consent was obtained from at least one of the parents or legal guardians. The study was approved by the ethics committee (Comité de Protection des Personnes (CPP)) Sud-Méditerranée II under the registration number (ID-RCB) 2017-A00510-53, dated 7 July 2017, and was conducted according to the recommendations of Good Clinical Practice and the Declaration of Helsinki.

At the time of enrolment, investigators recorded demographics, medical history, and physical examination data. Clinical variables evaluated at enrolment included: (i) signs of poor tolerance to fever (hemodynamics, neurological, respiratory), (ii) general appearance at clinical examination (good, intermediate, bad), and (iii) associated symptoms (meningeal syndrome, otolaryngologic symptoms, vomiting, diarrhea, skin rash, purpura, acute otitis media). Standard care, as per investigator assessment, included white blood cell count, absolute neutrophil count, blood culture, CRP and/or PCT levels, urinalysis, lumbar puncture, stool culture, ultrasound scanning, and chest radiography. The decision to hospitalise or treat the patient with antibiotics was left to the discretion of the physician. At day 7 post enrolment, a clinical follow-up was conducted, by phone for discharged patients or based on medical electronic records for hospitalised patients. 

In the absence of gold standard for bacterial and viral infections’ diagnosis, an expert panel reference standard was established, following existing recommendations [34]. This independent adjudication committee, composed of two panels of several infectious disease paediatricians, one microbiologist, and one methodologist expert, assigned one of six diagnosis categories: (i) proven bacterial infection, (ii) presumed bacterial infection, (iii) proven viral infection, (iv) presumed viral infection, (v) mixed infection (bacterial and viral co-infection), or (vi) unclassified fever. Classification by the adjudication committee was based on the clinical data collected at inclusion and at day 7, including antimicrobial treatment and hospitalisation decisions, but was blinded to all biomarker measurement results and to the decision of other members of the adjudication committee. Classification by the adjudication committee did not depend on age of patients. Clinical data comprised results of the clinical examination (at inclusion and at day 7), biological and microbiological laboratory tests, and medical imaging. Final diagnosis was determined by panel majority agreement. In case of non-majority, members of the two expert panels made a consensus decision on diagnosis.

### 2.2. Sample Collection and Biomarker Measurements

At inclusion, up to 3 mL blood was collected solely at the time of the venipuncture prescribed for standard care, for the dosage of the seven selected host-protein markers (CRP, PCT, MxA, TRAIL, NGAL, IP-10, IL-6). In case of venipuncture failure (when venipuncture could not be performed or blood volume was insufficient), the dosage of biomarkers was not performed and the patient was defined as having no biomarker data.

The dosage of the seven biomarkers was performed in a central laboratory (Joint Research Unit HCL-bioMérieux, Lyon, France), using serum (CRP, PCT, TRAIL, NGAL, IP-10, IL-6) or heparinised blood (MxA). Sera were prepared from 2 mL whole blood collected in BD Vacutainer Serum Separating Tubes II Advance Tube (Beckon Dickinson, BD366882, Le Pont-de-Claix, France). After 2 h clotting at room temperature and centrifugation at 2500× *g* for 10 min, sera were aliquoted and stored frozen at −80 °C until biomarkers’ measurements. Heparinised whole blood samples (500 µL) were collected in Microtainer tubes (Beckon Dickinson, BD365966, Le Pont-de-Claix, France) and stored frozen at −80 °C until MxA measurement.

PCT was measured in serum (200 µL) using the bioMérieux VIDAS B.R.A.H.M.S PCT assay (Marcy l’Etoile, France) and the VIDAS 30 instrument. CRP, TRAIL, IP-10, and IL-6 concentrations were measured in serum with the automated immunoassay Simple Plex platform (Protein simple, San Jose, CA, USA), in accordance with the instructions of the manufacturer. Simple Plex is an integrated immunoassay system that consists of a disposable microfluidic cartridge and an automated analyzer, the ELLA instrument. TRAIL, IP-10, and IL-6 quantitation were simultaneously performed in a multiplex cartridge format using 50 µL of two-fold diluted serum. CRP concentration was measured in a single analyte cartridge format using 1:2000 diluted serum. NGAL levels were measured by using the HNL bact ELISA kit (Diagnostic Development, Uppsala, Sweden) using 1:100 diluted serum, in accordance with the instructions of the manufacturer. All measurements were performed in duplicate per manual ELISA. MxA was measured by ELISA (BioVendor, Brno, Czech Republic) from heparinised whole blood diluted 1:10 in lysis buffer (BioVendor, Brno, Czech Republic) and lysed 30 min at room temperature, in accordance with the instructions of the manufacturer.

Paediatric healthy samples used for the comparative biomarker quantification of CRP, PCT, TRAIL, NGAL, IP-10, and IL-6 had leftover sera collected and provided by Eurofins Biomnis Sample Library (Lyon, France), based on the principle of non-opposition. Parents or legal guardians were informed of the possible use of leftover samples for research purposes when not expressing their opposition by the end of the legal retention period, in accordance with French regulations. Since paediatric healthy whole blood samples were not available, MxA was measured in whole blood of adult healthy volunteers, obtained from the national blood service (Etablissement Français du Sang [EFS], Lyon, France), based on the principle of non-opposition. 

### 2.3. Definitions

The primary endpoint was bacterial infection, defined by the adjudication committee as proven bacterial infection, presumed bacterial infection, or mixed (bacterial and viral) infection, as detailed in Appendix A. The primary aim of the study was to identify new biomarker combinations that can differentiate bacterial from viral infection with higher performance than CRP and/or PCT alone, in children younger than 3 years old suspected of SBI and admitted for standard medical care. Targeted performance criteria of biomarker combinations (i.e., superiority to CRP and/or PCT alone) were estimated based on the performance of CRP and PCT in the literature [2]. A combination of biomarkers was estimated to provide added medical value over the single markers CRP and PCT if: (i) the positive likelihood ratio (LR+) reached a minimum of 5.67, ideally ≥ 8.5 (increased probability of SBI diagnosis), and (ii) the negative likelihood ratio (LR–) reached a maximum of 0.5, ideally ≤ 0.3 (decreased probability of SBI diagnosis).

### 2.4. Statistical Analysis 

To identify and validate marker combinations, patients’ samples were divided into a training (TRAIN) and an independent validation (TEST) dataset. Sample size was calculated separately for the TRAIN and the TEST sets. For the TRAIN set, a minimum of 50 patients per category was considered necessary to build combination models. Assuming a prevalence of bacterial infection of 15%, a minimum of 333 patients was planned. For the TEST set, aiming for the LR+ and LR− performance values defined above, a sample size of 356 patients was calculated [35,36]. Considering 30% of patients with missing data due to venipuncture failure and/or incomplete clinical data, the total number of planned inclusions was increased from 689 to 985 patients. Since enrolment of patients took place over approximately two years, as well as to reflect the whole spectra of seasonal infections, it was decided that patients included during the first year would constitute the TRAIN set (corresponding approximately to two-thirds of the patients), while patients included during the second year would constitute the TEST set.

Patients without biomarker data or with fever not linked to an infection or from unknown origin (unclassified fever) were excluded from the analysis. Patients diagnosed with a mixed infection were assigned to the bacterial infection group for the analysis because they were clinically managed similarly. No cutoff was pre-specified for each investigated biomarker or for their combinations.

Quantitative variables were described by the median and interquartile range (IQR) and compared between groups using the Mann–Whitney U test. Categorical variables were described by the frequency and percentage of each modality, and compared between groups using chi-squared or Fisher tests. For each marker, the area under the receiver operating characteristic (ROC) curve (AUC) was calculated, with its 95% confidence interval (CI). Markers (CRP, PCT, MxA, TRAIL, NGAL, IP-10, IL-6) were then combined (up to four out of seven markers per model) by logistic regression on the TRAIN population. If necessary, logarithmic transformations of the markers were considered to fulfill the hypothesis of linearity of the linear predictor. The threshold with a LR− of at most 0.3 and the maximum LR+ value was chosen. Sensitivity, specificity, and positive and negative predictive values (PPV and NPV) associated with these thresholds were calculated, as well as the AUC of the model, and the AUC corrected for optimism (20-times 5-fold cross validation). Models obtained were then applied to the TEST population, with the thresholds estimated on the TRAIN population. Models were also applied separately to the TEST population according to the age group (7–91 days for the ≤3-month-old group and 92 days–36 months for the >3-month-old group). Comparisons of AUC between models were performed using the Delong test, whereas comparisons of LR between models were performed using a random logistic mixed effect model. 

Post hoc analyses were conducted using machine learning-based classifiers (see Appendix B) [37]. Modelling features included five clinical variables (signs of poor tolerance to fever (yes/no), general appearance (bad/intermediate/good), associated symptoms (yes/no), fever duration (<12 h/12—24 h/>24 h), and age) and four markers (CRP, PCT, MxA, TRAIL) in two-marker combinations (CRP/TRAIL, PCT/TRAIL, CRP/MxA, PCT/MxA, MxA/TRAIL). Classification models were built from the TRAIN dataset using the Caret package in R [37]. To avoid overfitting, the models were trained using 100 resampling according to the Leave Group Out Cross-Validation (LGOCV) method, also available in Caret. Performance was assessed using the independent validation (TEST) cohort. The AUC with a 95% CI was estimated for each dataset using the pROC package in R [38]. Sensitivity, specificity, PPV, NPV, LR+, and LR− were defined based on a decision threshold chosen to target a LR− of at most 0.3 and the maximum LR+. Analyses were conducted on the whole population, as well as per age group (≤ and >3 months of age). A subanalysis focusing on patients with proven bacterial infection was also conducted.

Statistical analyses were performed using SAS version 9.4 (SAS Institute, Cary, NC, USA) and R version 4.0.4 (The R Foundation, Vienna, Austria). *p*-values ≤ 0.05 were considered statistically significant.

## 3. Results

### 3.1. Patients’ Characteristics

**Figure 1:**
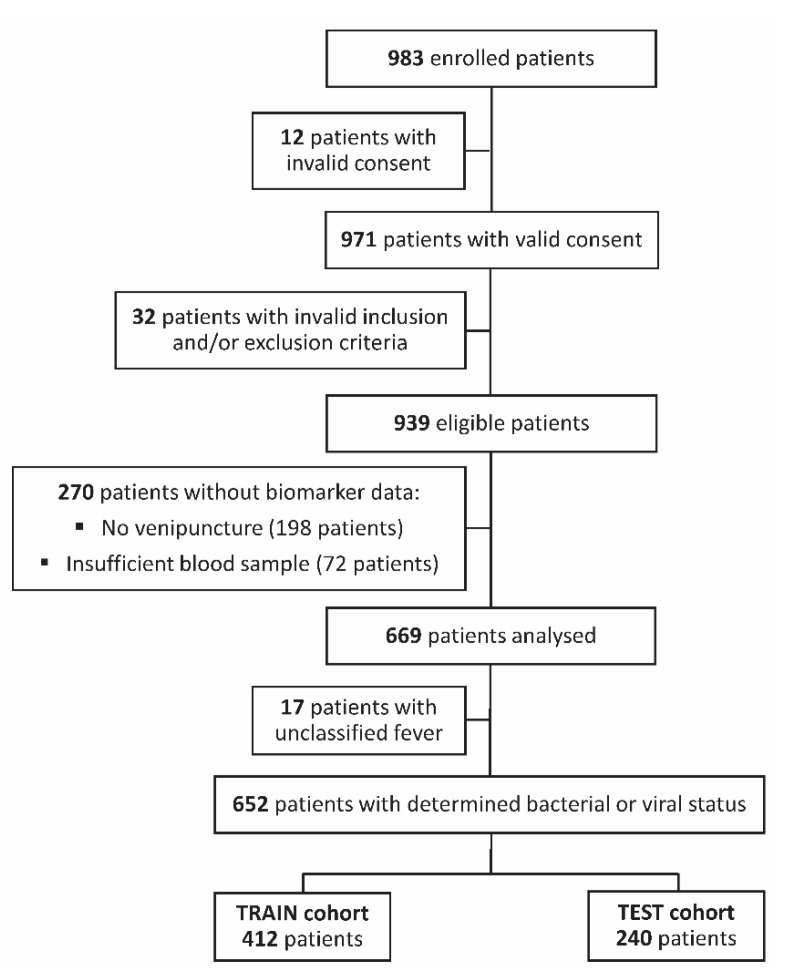
Study flow diagram. Out of 983 enrolled paediatric patients, 12 were ineligible due to invalid informed consent and 32 due to invalid inclusion and/or exclusion criteria. Out of the 939 eligible patients, 270 patients with missing biomarker data due to sampling failure were excluded from the analysis. Out of 669 analysed patients, 17 with indeterminate infection status (unclassified fever) were excluded from computational modelling. Altogether, 652 patients with a determined infection status were partitioned into a training (TRAIN) cohort (*n* = 412) and an independent validation (TEST) cohort (*n* = 240).

A total of 983 paediatric patients aged 7 days to 36 months with a suspected SBI were enrolled. Forty-four patients were excluded due to either invalid informed consent or invalid inclusion and/or exclusion criteria (Figure 1). Out of 939 eligible patients, 270 with missing biomarker data because of venipuncture failure were excluded from the analysis (Figure 1). Of the 669 analysed patients, 17 (2.6%) had an indeterminate infection status (unclassified fever), according to the expert adjudication committee (Figure 1 and Table 1). A total of 652 patients with a determined infection status were partitioned into a training cohort (TRAIN set; *n* = 412) for biomarker computational modelling and a validation cohort (TEST set; *n* = 240) for the confirmation of model performance on an independent cohort (Figure 1).

Out of the 669 analysed patients, 287 (42.9%) were hospitalised, including 279 (97.2%) in the paediatric ward, 7 (2.4%) in the intermediate care unit, and 1 (0.3%) in the intensive care unit. In total, 338/669 (50.5%) patients were subjected to medical imaging, including 201 (59.5%) chest radiography, of which 69 (34.3%) were abnormal; 142/338 (42.0%) patients with medical imaging received an ultrasound scanning, of which 49 (34.5%) suggested an infection; and 219/669 (32.7%) admitted children received an initial antibiotherapy. At the 7-day follow-up, 547/669 (81.8%) parents or legal guardians could be contacted. Apyrexia in the last 48 h was documented in 470 (70.2%) patients. On day 7, 108/331 (32.6%) outpatients received a new medical consultation, and 9 (2.7%) necessitated a secondary hospitalisation.

Among the 669 analysed patients, the adjudication committee diagnosed 123/422 (29.1%) and 83/247 (33.6%) bacterial infections and 289/422 (68.5%) and 157/247 (63.5%) viral infections in the TRAIN and TEST sets, respectively (Table 1; chi-squared test TRAIN vs. TEST sets, *p* = 0.427). This elevated rate of SBI in our cohort, compared to that generally reported in the general paediatric population, which ranges from 7% to 28% [1,2,3], might be due to the inclusion of children admitted to PED who required a clinical examination and were prescribed biological sample collection and analysis.

Patients’ characteristics in terms of sex, age, and clinical variables were comparable in the TRAIN and TEST sets (Table 2). Altogether, 163 pathogens were identified in 150 patients with proven infections (Table S1). The most prevalent detected bacterium was *E. coli*, and predominant viruses were picornaviruses, respiratory syncytial virus, and influenza virus (Table S1).

### 3.2. Biomarkers’ Characteristics

The concentrations of the seven investigated biomarkers in the sera (CRP, PCT, TRAIL, NGAL, IP-10, IL-6) or whole blood (MxA) of patients diagnosed with a bacterial vs. viral infection, in the TRAIN and TEST cohorts, are shown in Figure S1 and Table S2. A healthy cohort was tested as a reference. Marker concentrations according to the age class (≤3 months and >3 months of age) are shown in Figure S2 and Table S3.

### 3.3. Performance of Biomarker Combinations Using Logistic Regression

The seven selected biomarkers from the TRAIN set were combined, up to four markers, by logistic regression (Table S4). The number of markers per combination was limited to four, to remain amenable to clinical practice. The 98 trained models were then applied to the TEST set for an independent validation. Model performance was evaluated in terms of AUC, LR+, and LR− (Table S5). The targeted performance criteria were (i) a LR+ of minimum 5.67, ideally ≥ 8.5 (increased probability of SBI diagnosis), and (ii) a LR− of maximum 0.5, ideally ≤ 0.3 (decreased probability of SBI diagnosis). While models with LR− ≤ 0.3 were obtained, the maximum LR+ did not exceed 4.21 for a three-marker combination (model 57: NGAL/MxA/TRAIL) (Tables S5 and S6). Although the LR+ of model 57 was higher than that achieved by PCT (LR+ = 1.03) and CRP (LR+ = 2.22), the difference was not statistically significant (*p* = 0.677 and *p* = 0.884, respectively). The AUC (95% confidence interval (CI)) of model 57 was 0.831 (0.777–0.886).

**Figure 2:**
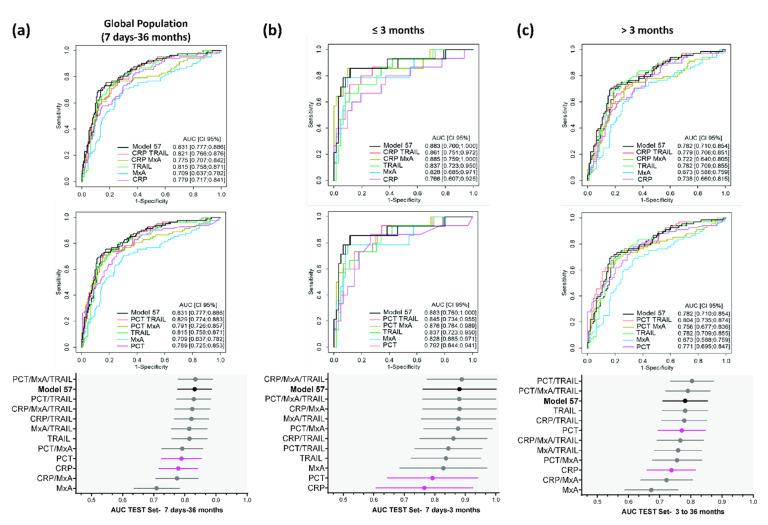
Performance of marker combinations using logistic regression (TEST set). ROC curves and forest plots of best performing models are shown for the global population (**a**), the ≤ 3-month-old patients (**b**), and the > 3-month-old patients (**c**). The upper ROC curves show the comparisons to CRP (pink curves) and the lower ROC curves the comparisons to PCT (pink curves). The forest plots depict AUC (plain circles) and respective 95% CI (horizontal whiskers). Model 57 (NGAL/MxA/TRAIL; black curves, black whiskers) performs best in terms of LR+ in the global population. Complete performance results of the depicted models are shown in Table S6. Performance results of all 98 models are shown in Table S5. The AUC of model 57 (0.831) was not statistically different from that of PCT (0.789; *p* = 0.115) but was significantly better than that of CRP (0.779; *p* = 0.042). The 4-marker model PCT/NGAL/MxA/TRAIL (model 67) achieved the highest AUC (0.839; Table S5). The difference in AUC of model 67 to that of PCT was close to statistical significance (*p* = 0.053), while that of model 67 vs. CRP was statistically significant (*p* = 0.014).

Importantly, model performances were consistent between the TRAIN and TEST set, with the top five models in the TEST set being among the top nine models in the TRAIN set (Tables S4 and S5). As for the contribution of individual markers, IL-6 did not improve the performance of PCT or CRP, and IP-10 and NGAL did not improve the performance of CRP and only slightly improved the performance of PCT. Altogether, IL-6, IP-10, and NGAL minimally improved the performance of the associated models (Table S5). On the other hand, while MxA did not improve the performance of PCT or CRP, TRAIL alone did improve their performance. Interestingly, the MxA/TRAIL pair showed a reasonably good performance (model 22: top 16 with AUC = 0.814, LR+ = 2.85, LR− = 0.32), which was further increased in association with CRP (model 52: top 11 with AUC = 0.824, LR+ = 3.28, LR− = 0.32) or PCT (model 63: top 9 with AUC = 0.834, LR+ = 3.29, LR− = 0.3) (Table S5). Notably, the top 13 combinations in the global population all included the MxA/TRAIL pair (both in the TRAIN and TEST sets) (Tables S4 and S5). Figure 2a depicts the representative ROC curves and AUC forest plots, showing model 57 (black) in comparison to PCT and CRP alone (pink) as well as models including MxA and TRAIL. Additional performance values are shown in Table S6.

Interestingly, application of these models to the two age class populations (≤ and >3 months) revealed better performances (higher LR+) in the younger patients’ group (7–91 days of age; *n* = 77). Out of the 98 generated models, 17 fulfilled the targeted performance criteria in the 7–91-day-old group, of which 15 included the MxA/TRAIL pair (Table S5). AUC estimates were also globally higher in the ≤ 3-month-old group, although the respective 95% CIs were broader (Figure 2b vs. Figure 2c and Table S6). Regarding the AUC and LR estimates (and the number of contributing markers), the best-performing model in the ≤3-month population was CRP/MxA/TRAIL (model 52; AUC = 0.895, LR+ = 10.46, LR− = 0.16) (Tables S5 and S6).

Altogether, the combination of up to four out of seven biomarkers by logistic regression allowed for reaching the predefined performance criteria in younger patients (7–91 days of age) but not in the global population (7 days–36 months). The best marker combinations included the viral biomarkers MxA and TRAIL. 

### 3.4. Performance of Combinations of Clinical Variables and Biomarkers Using Machine Learning (Post Hoc Analysis)

To identify models discriminating bacterial from viral infection more accurately, notably in the 7 days–36 months population, a post hoc analysis was conducted combining relevant clinical variables with a minimal set of biomarkers and using machine learning-based modelling. Models integrating clinical parameters and biomarkers have indeed proved to be more performant than clinical parameters or biomarkers alone [1,5,6,7,23]. The analysis was limited to two-marker combinations, to keep the approach practicable in clinical routine. Given their good performance in the previous approach, MxA and TRAIL were selected in addition to CRP and PCT and were tested in the following combinations: CRP/TRAIL, PCT/TRAIL, CRP/MxA, PCT/MxA, and MxA/TRAIL. Five clinical variables (5CV) normally applied during clinical examination at the participating PED for the diagnosis of SBI were selected by the clinicians as modelling features: signs of poor tolerance to fever, general appearance, associated symptoms, fever duration, and age (Table 2).

**Figure 3:**
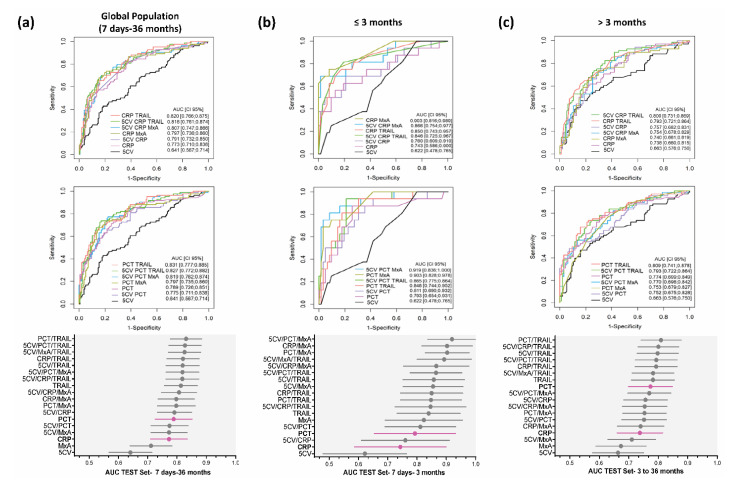
Performance of models combining biomarkers and clinical variables using machine learning (TEST set). ROC curves and forest plots of best performing models are shown for the global population (**a**), the ≤3-month-old patients (**b**), and the >3-month-old patients (**c**). The upper ROC curves show the comparisons to CRP (pink curves) and the lower ROC curves the comparisons to PCT (pink curves). The clinical variables alone (5CV) are shown as black curves. The forest plots depict AUC (plain circles) and respective 95% CI (horizontal whiskers). Complete performance results are shown in Table S7. In terms of AUC, PCT/TRAIL performed best in the global population, with an AUC of 0.831. The difference in AUC between PCT/TRAIL and PCT (AUC = 0.789) was close to statistically significant (*p* = 0.056), while that between PCT/TRAIL and CRP (AUC = 0.773) was statistically significant (*p* = 0.025). In the ≤3-month-old group, the best model in respect of both AUC and LR was 5CV/PCT/MxA (AUC = 0.919, LR+ = 21.31, LR− = 0.32). The differences in AUC between 5CV/PCT/MxA and PCT (AUC = 0.793) and between 5CV/PCT/MxA and CRP (AUC = 0.743) were both statistically significant (*p* = 0.030 and *p* = 0.005, respectively).

**Figure 4:**
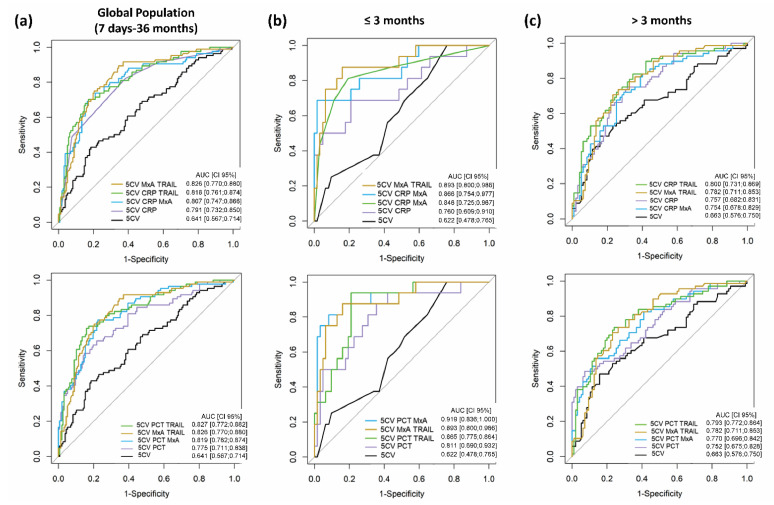
Performance of machine learning-based models including MxA and TRAIL together with clinical variables (TEST set). ROC curves and forest plots of best performing models including MxA and TRAIL are shown for the global population (**a**), the ≤ 3-month-old patients (**b**), and the >3-month-old patients (**c**). The upper ROC curves show the combinations with CRP and the lower ROC curves the combinations with PCT. The bad-performing model including clinical variables only (5CV) is depicted in black, and the good-performing model (5CV/MxA/TRAIL) is depicted in brown. Respective forest plots are shown in Figure 3. Complete performance results are shown in Table S7.

The targeted performance criteria (LR− ≤ 0.3, maximum LR+) and the training and validation sets were the same as in the previous approach. Although the performance of machine learning-based classifiers generated by the combination of the 5CV and biomarkers was better than that of the 5CV and biomarkers alone, the classifiers failed to reach the aimed performance criteria in the global population, with a maximum LR+ of 3.62 (LR− = 0.33; AUC (95% CI) = 0.826 (0.770–0.880)) for the combination 5CV/MxA/TRAIL (Table S7, Figure 3a and Figure 4a). As observed before, model performances were greater in younger (7–91-day-old) patients. The top five models in this group fulfilled the aimed performance criteria (Table S7). The best model in respect to both AUC and LR in the ≤3-month-old group was the combination of 5CV/PCT/MxA (AUC (95% CI) = 0.919 (0.770–0.880), LR+ = 21.31, LR− = 0.32) (Table S7, Figure 3b,c and Figure 4b,c). 

Finally, the validity of the machine learning approach was verified on the group of proven bacterial and viral infections within the global population (TRAIN set, *n* = 86; TEST set, *n* = 64; see Table 1). As anticipated, higher performances were observed, with the highest AUC for 5CV/CRP/MxA (AUC (95% CI) = 0.944 (0.883–1.000)). The best model for both AUC and LR was for 5CV/CRP/TRAIL (AUC (95% CI) = 0.937 (0.882–0.991), LR+ = 20.43, LR− = 0.25) (Figure S3).

### 3.5. Performance of the Best Models for Antibiotherapy Management 

The performances of the best models generated by logistic regression and machine learning were next evaluated regarding antibiotherapy management, using the classification by the expert adjudication committee as the reference standard. For 214/240 (89.2%) patients of the validation cohort, the applied antibiotherapy protocol agreed with the classification of a bacterial or viral infection by the adjudication committee (i.e., treated bacterial infections and untreated viral infections) (Table 3). For 26/240 (10.8%) patients, the antibiotherapy decision disagreed with the classification by the adjudication committee, with 11 untreated patients classified as having a bacterial infection and 15 treated patients classified as having a viral infection (Table 3). Importantly, none of the 11 untreated SBI had developed clinical complications at the 7-day follow-up.

Reclassification by selected models of the infection status of the 26 discordant and 214 concordant classifications of the adjudication committee is shown in Table 4. While single markers, in particular PCT, performed well to rule in SBI (10/11 (90.9%) untreated SBI reclassified as SBI and only 4/72 (5.6%) treated SBI wrongly reclassified as non-SBI by PCT), they were poor in ruling it out (128/140 (91.4%) untreated non-SBI reclassified as SBI by PCT). By contrast, models tended to better rule out SBI, while showing some benefit in ruling it in (Table 4). The benefit of these models is, however, mitigated by the observation that only one untreated pneumococcus infection would have been correctly classified as SBI by the best models, and a few “untreated SBI reclassified as SBI” (2/8 for NGAL/MxA/TRAIL, 1/9 for 5CV/MxA/TRAIL) were salmonella infections in older (2 and 3 years of age) children, for which no antibiotics therapy would have been recommended at our PED. In addition, one case of untreated bacterial and viral co-infection was never correctly reclassified as SBI by the best three models (NGAL/MxA/TRAIL, 5CV/PCT/TRAIL, 5CV/MxA/TRAIL).

## 4. Discussion

This prospective, multicentre, observational study evaluated the potential benefit of combining bacterial- and viral-induced host-protein biomarkers to improve the diagnosis of SBI in febrile children aged 7 days to 36 months.

Using logistic regression applied to blood levels of selected protein markers, we showed that combinations of biomarkers (with or without selected clinical variables) performed slightly better than CRP or PCT alone to diagnose SBI. However, the poor LR+ and acceptable LR− observed in the 7 days–36 months population indicate that these models might be better to rule out SBI, rather than ruling it in. The small improvement of the best generated models over CRP and PCT was confirmed by the reclassification of patients that showed a discordance between the infection status assigned by the adjudication committee and the decision to treat with antibiotics by the physician. Notably, compared to single markers and especially to PCT, the models tended to better rule out SBI, as previously reported by other modelling approaches [1,5,8,9,10]. Nonetheless, the benefit of this reclassification was marginal, first because the concordance rate of SBI diagnosis by the adjudication committee with antibiotherapy was already high (89.2%) and second because the few cases of SBI diagnosis not treated with antibiotics might have benefited from antibiotherapy. This demonstrates the good clinical performance of SBI diagnosis by the standard of care in this study. Nevertheless, the validity of our computational approach was supported by the better diagnostic performance achieved in the subgroup of patients with proven bacterial infection (e.g., LR+ = 20.43, LR− = 0.25 for 5CV/CRP/TRAIL; post hoc analysis). Importantly, satisfactory LR+ performances were also obtained when focusing on the ≤ 3-months population (e.g., LR+ = 10.46, LR− = 0.16 for CRP/MxA/TRAIL; LR+ = 9.3, LR− = 0.27 for 5CV/MxA/TRAIL), suggesting that our approach might mainly benefit this younger age class. As the size of this age group is small in our study (*n* = 107 in TRAIN set, *n* = 77 in TEST set; Table 2), our observations must be verified in a large cohort study of ≤3-month-old febrile patients. If confirmed, our modelling approach would distinguish itself from previously reported clinical prediction models showing similar performances in patients aged < 3 months and 3–12 months [7].

Our logistic regression approach confirmed previous studies showing that marker combinations perform better than single markers and that a combination of bacterial and viral biomarkers can improve the classification of SBI [1,11,16,22,24,25]. Indeed, most top combinations consisted of both bacterial- and viral-induced markers. Surprisingly, the viral markers MxA and TRAIL performed best as a two-marker combination, and the pair MxA/TRAIL was part of most of the best performing models. The good performance of MxA/TRAIL was particularly evident in the ≤3 months group (LR+ = 7.99, LR− = 0.24), which was further increased in association with the bacterial-induced markers PCT (LR+ = 9.43, LR− = 0.23) and CRP (LR+ = 10.46, LR− = 0.16). Why the two viral-induced markers MxA and TRAIL performed well in combination for the diagnosis of SBI in younger febrile patients remains to be investigated.

The ImmunoXpert host-signature assay computationally integrates the blood levels of CRP, TRAIL, and IP-10 [22,24,25,29]. As opposed to our approach, the ImmunoXpert assay is based on predefined cutoffs. In our study, the CRP/TRAIL/IP-10 combination (model 53) performed worse than CRP alone or CRP/TRAIL (model 27), both in the global population and in patients ≤3 months of age (Table S5). In patients >3 months of age, CRP/TRAIL/IP-10 performed comparably to CRP and worse than CRP/TRAIL (Table S5). Similarly, the FebriDx rapid test integrates blood levels of CRP and MxA based on predefined cutoffs [26]. In our study, the CRP/MxA combination (model 20) performed worse than CRP alone in both the global and ≤3-month-old populations. These observations demonstrate the impact of the modelling and scoring method on the potential value of biomarkers. In the case of the ImmunoXpert assay, the definition of the age group might also impact the diagnosis outcome, as these studies included older paediatric patients compared to our study [22,24,25,29]. The difference between studies in the definition of bacterial infection and SBI, due to the lack of a validated and standardised risk stratification tool, might also influence the results.

Our study presents several strengths and limitations. The major strengths are the large size of the young children enrolled in the study (*n* = 983) and the real-life setting including proven, presumed, and mixed infections. Other strengths are the double-blinded study design (investigator blinded to biomarker results, adjudication committee blinded to biomarker results and to routine PCT and/or CRP results possibly ordered by the physician), and the multicentre design involving three French PED. As for the computational analysis, a key strength is the definition of a training cohort for model generation and an independent test cohort for model validation. Limitations include possible heterogeneity in patient management between centres, although this might be considered a strength, as it better reflects the real-life clinical setting. Another possible limitation is the management of patients suspected of SBI per investigator assessment, which might have introduced interpatient heterogeneity, albeit again reflecting the current real-life clinical setting. Future biomarker studies would surely benefit from a standardised phenotyping and risk stratification tool for children with suspected SBI [6]. Next, the adjudication panel might have been influenced in their diagnosis assignment by the knowledge of the antibiotherapy decision of the investigator, thus potentially introducing a bias in the definition of the SBI group. In addition, the predefined age class (7 days–36 months) might have been too broad, probably not accurately reflecting the differences in the clinical setting between younger (≤3 months) and older (>3 months) children. This is supported by the better model performances observed in younger children in our study. Finally, the seasonal (rather than random) assignment of the training and validation cohorts was epidemiologically justified but might have introduced a time-dependent bias in the analysis.

## 5. Conclusions

Although our study failed to identify marker combinations outperforming CRP or PCT alone in 7 days–36 months febrile children, it confirmed the benefit of integrating bacterial- and viral-induced host biomarkers in computationally generated prediction models; this appeared to be especially the case for ≤3-month-old febrile patients. Refinement of this approach will be needed to significantly improve prediction tools. The limited increase in performance, achieved by selecting well-known markers of bacterial and viral infections, also suggests that clinical decision rules (e.g., Lab-score, Step-by-Step approach) [1,3,5,6,7] might be more adapted to clinical practice, or that alternative, unbiased proteomics or transcriptomics approaches might be more powerful to identify novel SBI biomarkers more accurately, thereby better assisting paediatricians in the diagnosis and management of febrile young children.

## Figures and Tables

**Table 1:** Infection classification in the whole, training (TRAIN), and validation (TEST) cohorts.

Infection Class		Whole Cohort (*n* = 669) N (%)	TRAIN Set (*n* = 422) N (%)	TEST Set (*n* = 247) N (%)
**Bacterial infection**	Proven	90 (13.4)	53 (12.6)	37 (15.0)
	Presumed	71 (10.6)	50 (11.8)	21 (8.5)
	Mixed ^1^	45 (6.7)	20 (4.7)	25 (10.1)
	Total	206 (30.8)	123 (29.1)	83 (33.6)
**Viral infection**	Proven	60 (8.9)	33 (7.8)	27 (10.9)
	Presumed	386 (58.0)	256 (60.7)	130 (52.6)
	Total	446 (66.7)	289 (68.5)	157 (63.5)
**Unclassified fever ** ^2^	Total	17 (2.6)	10 (2.4)	7 (2.8)

^1^ Mixed (bacterial and viral) infections were assigned to the bacterial infection group because they were clinically managed similarly; ^2^ patients with unclassified fever were excluded from the TRAIN and TEST sets for the computational analysis. A chi-squared test showed no statistically significant difference in infection classification between the TRAIN and TEST sets (*p* = 0.427).

**Table 2:** Patients’ characteristics in TRAIN and TEST sets, according to the viral or bacterial infection status.

	TRAIN Set			TEST Set		
Study Population According to Infection Status	Viral (N = 289)	Bacterial (N = 123)	Total (N = 412)	Viral (N = 157)	Bacterial (N = 83)	Total (N = 240)
Sex, N (%)						
Female	132 (45.7%)	55 (44.7%)	187 (45.4%)	74 (47.1%)	43 (51.8%)	117 (48.8%)
Male	157 (54.3%)	68 (55.3%)	225 (54.6%)	83 (52.9%)	40 (48.2%)	123 (51.2%)
Age in days						
Median	335.0	393.0	345.0	169.0	403.0	264.5
Range	8.0–1080.0	10.0–1094.0	8.0–1094.0	12.0–1084.0	13.0–1075.0	12.0–1084.0
Interquartile range	85.0–570.0	133.5–571.5	88.7–571.2	71.0–571.0	132.5–690.0	78.0–603.0
Age class, N (%)						
≤3 months	82 (28.4%)	25 (20.3%)	107 (26.0%)	62 (39.5%)	15 (18.1%)	77 (32.1%)
>3 months	207 (71.6%)	98 (79.7%)	305 (74.0%)	95 (60.5%)	68 (81.9%)	163 (67.9%)
Season, N (%)						
Winter	77 (26.6%)	22 (17.9%)	99 (24.0%)	37 (23.6%)	20 (24.1%)	57 (23.8%)
Spring	50 (17.3%)	28 (22.8%)	78 (18.9%)	26 (16.6%)	18 (21.7%)	44 (18.3%)
Summer	63 (21.8%)	28 (22.8%)	91 (22.1%)	41 (26.1%)	19 (22.9%)	60 (25.0%)
Autumn	99 (34.3%)	45 (36.6%)	144 (35.0%)	53 (33.8%)	26 (31.3%)	79 (32.9%)
General appearance at clinical examination, N (%)						
Good	161 (55.7%)	59 (48.0%)	220 (53.4%)	85 (54.1%)	37 (44.6%)	122 (50.8%)
Intermediate	74 (25.6%)	41 (33.3%)	115 (27.9%)	42 (26.8%)	31 (37.3%)	73 (30.4%)
Bad	10 (3.5%)	3 (2.4%)	13 (3.2%)	6 (3.8%)	4 (4.8%)	10 (4.2%)
Unknown	44 (15.2%)	20 (16.3%)	64 (15.5%)	24 (15.3%)	11 (13.3%)	35 (14.6%)
Associated symptoms at clinical examination, N (%) ^1^						
No	58 (20.1%)	49 (39.8%)	107 (26.0%)	41 (26.1%)	29 (34.9%)	70 (29.2%)
Yes	229 (79.2%)	74 (60.2%)	303 (73.5%)	110 (70.1%)	54 (65.1%)	164 (68.3%)
Unknown	2 (0.7%)	0 (0.0%)	2 (0.5%)	6 (3.8%)	0 (0.0%)	6 (2.5%)
Signs of poor tolerance to fever, N (%) ^2^						
No	216 (74.7%)	104 (84.6%)	320 (77.7%)	114 (72.6%)	69 (83.1%)	183 (76,2%)
Yes	71 (24.6%)	17 (13.8%)	88 (21.3%)	38 (24.2%)	14 (16.9%)	52 (21.7%)
Unknown	2 (0.7%)	2 (1.6%)	4 (1.0%)	5 (3.2%)	0 (0.0%)	5 (2.1%)
Fever duration, N (%)						
<12 h	59 (20.4%)	14 (11.4%)	73 (17.7%)	47 (29.9%)	14 (16.9%)	61 (25.4%)
12–24 h	76 (26.3%)	28 (22.8%)	104 (25.3%)	34 (21.7%)	21 (25.3%)	55 (22.9%)
>24 h	153 (52.9%)	81 (65.8%)	234 (56.8%)	75 (47.8%)	48 (57.8%)	123 (51.3%)
Unknown	1 (0.4%)	0 (0.0%)	1 (0.2%)	1 (0.6%)	0 (0.0%)	1 (0.4%)

^1^ meningeal syndrome, otolaryngologic symptoms, vomiting, diarrhea, skin rash, purpura, and acute otitis media; ^2^ hemodynamics, neurological, and/or respiratory symptoms.

**Table 3:** Concordance between the classification by the adjudication committee and antibiotherapy (TEST set; *n* = 240).

		Treatment with Antibiotics	
		Yes	No
**Bacterial infection**	Yes	72	11
	No	15	142

**Table 4:** Reclassification of serious bacterial infection (SBI) by the best models (TEST set; *n* = 240).

Selected Models	Classified as SBI ^3^		Classified as Non-SBI ^3^	
	Treated SBI Reclassified as Non-SBI (*n*/N, %)	Untreated SBI Reclassified as SBI (*n*/N, %) ^4^	Treated Non-SBI Reclassified as Non-SBI (*n*/N, %)	Untreated Non-SBI Reclassified as SBI (*n*/N, %) ^5^
PCT ^1^	4/72 (5.6%)	10/11(90.9%)	2/15 (13.3%)	128/140 (91.4%)
CRP ^1^	12/72 (16.7%)	8/11 (72.7%)	7/15 (46.7%)	50/142 (35.2%)
PCT/MxA/TRAIL ^1^	18/72 (25.0%)	9/10 (90.0%)	8/15 (53.3%)	29/139 (20.9%)
CRP/MxA/TRAIL ^1^	17/72 (23.6%)	7/10 (70.0%)	8/15 (53.3%)	29/141 (20.6%)
NGAL/MxA/TRAIL (model 57) ^1^	18/72 (25.0%)	8/10 (80.0%)	8/15 (53.3%)	21/141 (14.9%)
5CV/PCT/TRAIL ^2^	17/72 (23.6%)	7/11 (63.6%)	9/15 (60.0%)	29/142 (20.4%)
5CV/MxA/TRAIL ^2^	19/72 (26.4%)	9/11 (81.8%)	8/15 (53.3%)	25/142 (17.6%)

^1^ Logistic regression-based models; ^2^ machine learning-based models; ^3^ by the expert adjudication committee; ^4^ denominator <11 in some cases for logistic-regression-based models, due to one patient with missing biomarker data; ^5^ denominator <142 in some cases for logistic-regression-based models, due to several patients with missing biomarker data. Abbreviations: SBI, serious bacterial infection; 5CV, 5 clinical variables selected for computational modelling.

## Data Availability

The data presented in this study are available within the article or Supplementary Materials.

## Supplementary Materials

The following supporting information can be downloaded at: https://www.mdpi.com/article/10.3390/jcm11216563/s1. Figure S1: Biomarker levels in the global population (7 days–36 months) according to the bacterial and viral infections’ status, in comparison to healthy donors; Figure S2: Biomarker levels in the global population (7 days–36 months) and per age category (≤ and >3 months), according to the bacterial and viral infections’ status; Figure S3: Performance of machine learning-based models combining biomarkers and clinical variables in the 7 days–36 months cohort with proven bacterial and viral infections (TEST set; n = 64); Table S1: Identified pathogens in patients with a proven infection (N = 150); Table S2: Biomarker levels according to the bacterial and viral infections’ status, in comparison to healthy donors; Table S3: Biomarker levels according to the bacterial and viral infections’ status, in the global population (7 days–36 months) and per age category; Table S4: Performances of marker combinations using logistic regression (TRAIN set; n = 412); Table S5: Performances of marker combinations using logistic regression (TEST set; parameters estimated at the threshold of the TRAIN cohort); Table S6: Performances of top marker combinations using logistic regression (TEST set); Table S7: Performances of marker and clinical variable combinations using machine learning (TEST set).

## Appendix A. Bacterial and Viral Infection Definitions

The primary endpoint was bacterial infection, defined by the adjudication committee as a proven bacterial infection, a presumed bacterial infection, or a mixed (bacterial and viral) infection. SBI, viral infection, and unclassified fever were defined as follows:

### Appendix A.1. Serious Bacterial Infection (SBI)

- Proven SBI was established in the case of:
  - A positive Streptotest result
  - Bacteremia: pathogen detection in blood culture. A blood culture was considered negative if symptoms improved without antibiotherapy in the case of saprophytic germs (i.e., Staphylococcus coagulase-negative) or if the isolated germ was nonpathogenic (i.e., diphteroids, nonpathogenic neisseria, α-hemolytic streptococcus, etc.)
  - Bacterial meningitis: pleiocytosis (≥5 cells/mm^3^) and pathogen detection (culture-positive) in the cerebrospinal fluid. In the case of a hemorrhagic cerebrospinal fluid, only a positive culture for a pathogenic germ confirmed the diagnosis of proven SBI.
  - Bacterial urinary tract infection: established from a sample collected by urinary catheterisation or from a midstream specimen of urine (MSU), with a cytobacteriological examination showing white blood cells ≥10/mm^3^ and a pathogenic monomorphic flora ≥10^5^ CFU/mL.
  - Bacterial respiratory infection: radiological and clinical pneumonia associated with a rapid improvement under antibiotherapy.
  - Acute bacterial gastroenteritis: liquid stool and stool culture positive for a pathogenic germ.
  - Bacterial infection of soft tissue, cellulitis, or abscess: clinical diagnosis based on evidence of inflammatory skin changes or abscess. When possible, a microbiological collection was carried out, and the detection of a pathogenic germ confirmed the diagnosis of proven SBI.
  - Bacterial osteoarticular infection: positive culture of the fluid of an articular puncture.
- Presumed SBI was established in the case of:
  - Urinary tract infection: established from a sample collected from a urine bag (as opposed to urinary catheterisation; see proven SBI above)
- Mixed (bacterial and viral) infection was established in the case of patients who met the criteria for a proven or presumed bacterial infection and whose clinical or para-clinical data suggested a viral co-infection. Mixed infections were clinically managed like bacterial infections.

### Appendix A.2. Viral Infection

- Proven viral infection was established in the case of:
  - Viral meningitis: pleiocytosis (≥5 cells/mm^3^) and virus detection by PCR in the cerebrospinal fluid. In the case of a hemorrhagic cerebrospinal fluid, only virus detection confirmed the diagnosis of proven viral infection.
  - Respiratory syncytial virus (RSV) infection: respiratory or nasopharyngeal symptoms associated with a RSV-positive sample.
  - Influenza virus infection: detection of the virus in a nasopharyngeal sample.
  - Other viral respiratory infections: respiratory or nasopharyngeal symptoms associated with the detection of a virus other than those mentioned above.
  - Acute viral gastroenteritis: liquid stool and a stool that tested positive for a gastrointestinal virus.
- Presumed viral infection was established in the case of:
  - Presumed viral meningitis: pleiocytosis (≥5 cells/mm^3^) without evidence of a pathogenic bacteria (culture-negative) or of a virus (PCR-negative) in the cerebrospinal fluid. In the case of a hemorrhagic cerebrospinal fluid, only the evidence of a negative culture and PCR confirmed the diagnosis of presumed viral infection.
  - Rhinitis: only nasal discharge without evidence of a microbiological agent.
  - Nasopharyngitis: pharyngitis and nasal discharge without evidence of a microbiological agent.
  - Varicella zoster virus infection: based on clinical diagnosis.

### Appendix A.3. Unclassified Fever

- Fever of infectious origin without an identified cause: fever without clinical evidence of an infection site, for which all microbiological investigations were negative and the clinical course did not allow for the adjudication committee to assign a diagnosis.
- Fever of non-infectious origin: inflammatory fever (e.g., Kawasaki or Still’s disease).

## Appendix B. Computational Modelling Methods Used in Machine Learning

Machine learning-based classifiers were built on the TRAIN dataset using the Caret package in R [37]. To avoid overfitting, the models were trained using 100 resampling according to the Leave Group Out Cross-Validation (LGOCV) method available in Caret. The models available in the Caret package (https://topepo.github.io/caret/available-models; accessed on 10 October 2022) used in this study are listed in Table A1.

**Table A1:** Models of the Caret package used for machine learning in this study.

Model	Method Value	Type	Libraries	Tuning Parameters
eXtreme Gradient Boosting	xgbLinear	Classification, Regression	xgboost	nrounds, lambda, alpha, eta
Linear Discriminant Analysis	lda	Classification	MASS	None
Stochastic Gradient Boosting	gbm	Classification, Regression	gbm, plyr	n.trees, interaction.depth, shrinkage, n.minobsinnode
Generalized Additive Model using Splines	gam	Classification, Regression	mgcv	select, method
Random Forest	rf	Classification, Regression	randomForest	mtry
glmnet	glmnet	Classification, Regression	glmnet, Matrix	alpha, lambda
Bayesian Generalized Linear Model	bayesglm	Classification, Regression	arm	None
Generalized Linear Model	glm	Classification, Regression	-	None
Penalized Multinomial Regression	multinom	Classification	nnet	decay
Generalized Additive Model using LOESS	gamLoess	Classification, Regression	gam	span, degree
Boosted Generalized Linear Model	glmboost	Classification, Regression	plyr, mboost	mstop, prune
Conditional Inference Tree	ctree	Classification, Regression	party	mincriterion
Partial Least Squares	pls	Classification, Regression	pls	ncomp
Stabilized Linear Discriminant Analysis	slda	Classification	ipred	None
k-Nearest Neighbors	knn	Classification, Regression	-	k
Linear Distance Weighted Discrimination	dwdLinear	Classification	kerndwd	lambda, qval
