# Supplementary material for: Performance Evaluation of Host Biomarker Combinations for the Diagnosis of Serious Bacterial Infection in Young Febrile Children: A Double-Blind, Multicentre, Observational Study
Source: J Clin Med. 2022 Nov 4;11(21):6563. doi: 10.3390/jcm11216563 (PMC9657860; doi:10.3390/jcm11216563)
Supplement: Supplementary file 1 [file jcm-11-06563-s001.zip › Supplementary Materials/Figures S1-S3 & Tables S1-S3.pdf]

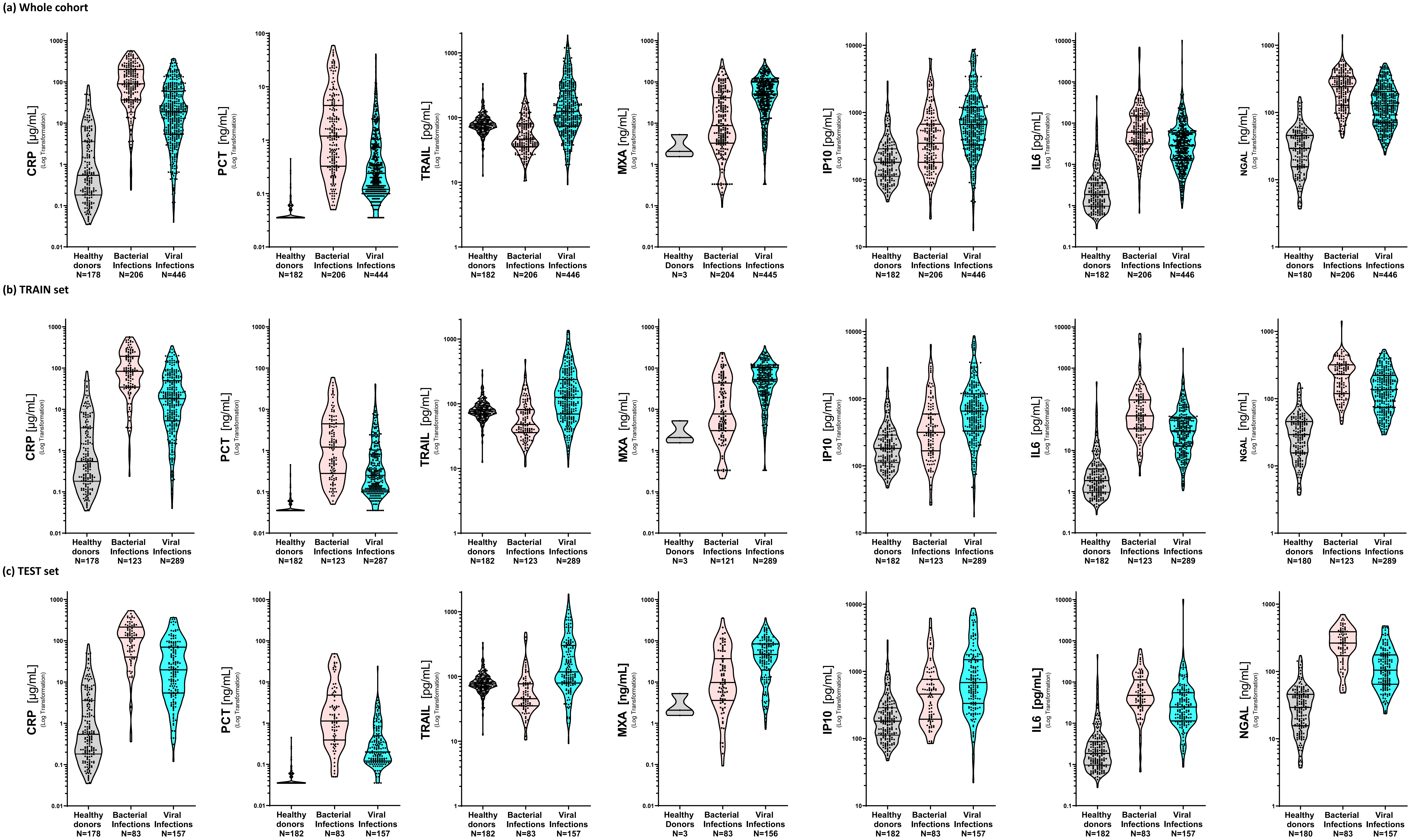

Figure S1. Biomarker levels in the global population (7 days – 36 months) according to the bacterial and viral infection status, in comparison to healthy donors. The concentration of the seven investigated biomarkers was measured as described in Materials and Methods. Distributions of the biomarker levels in the indicated populations (whole, TRAIN and TEST cohorts) are shown as violin plots. Respective median and interquartile range values are shown in Table S2.

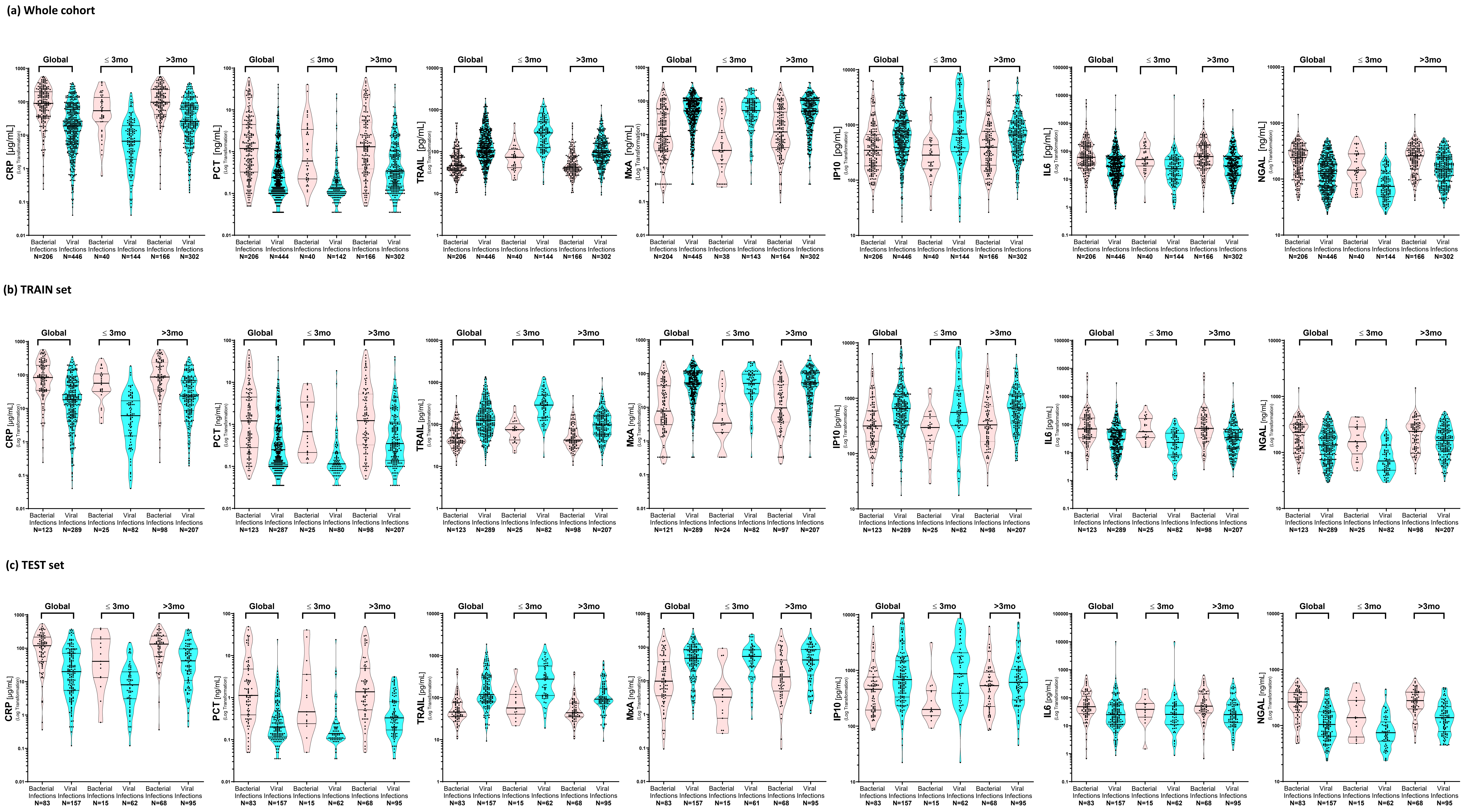

Figure S2. Biomarker levels in the global population (7 days – 36 months) and per age category (≤ and > 3 months), according to the bacterial and viral infection status. The concentration of the seven investigated biomarkers was measured as described in Materials and Methods. Distributions of the biomarker levels in the indicated populations (whole, TRAIN and TEST cohorts) are shown as violin plots. Respective median and interquartile range values are shown in Table S3.

(a)

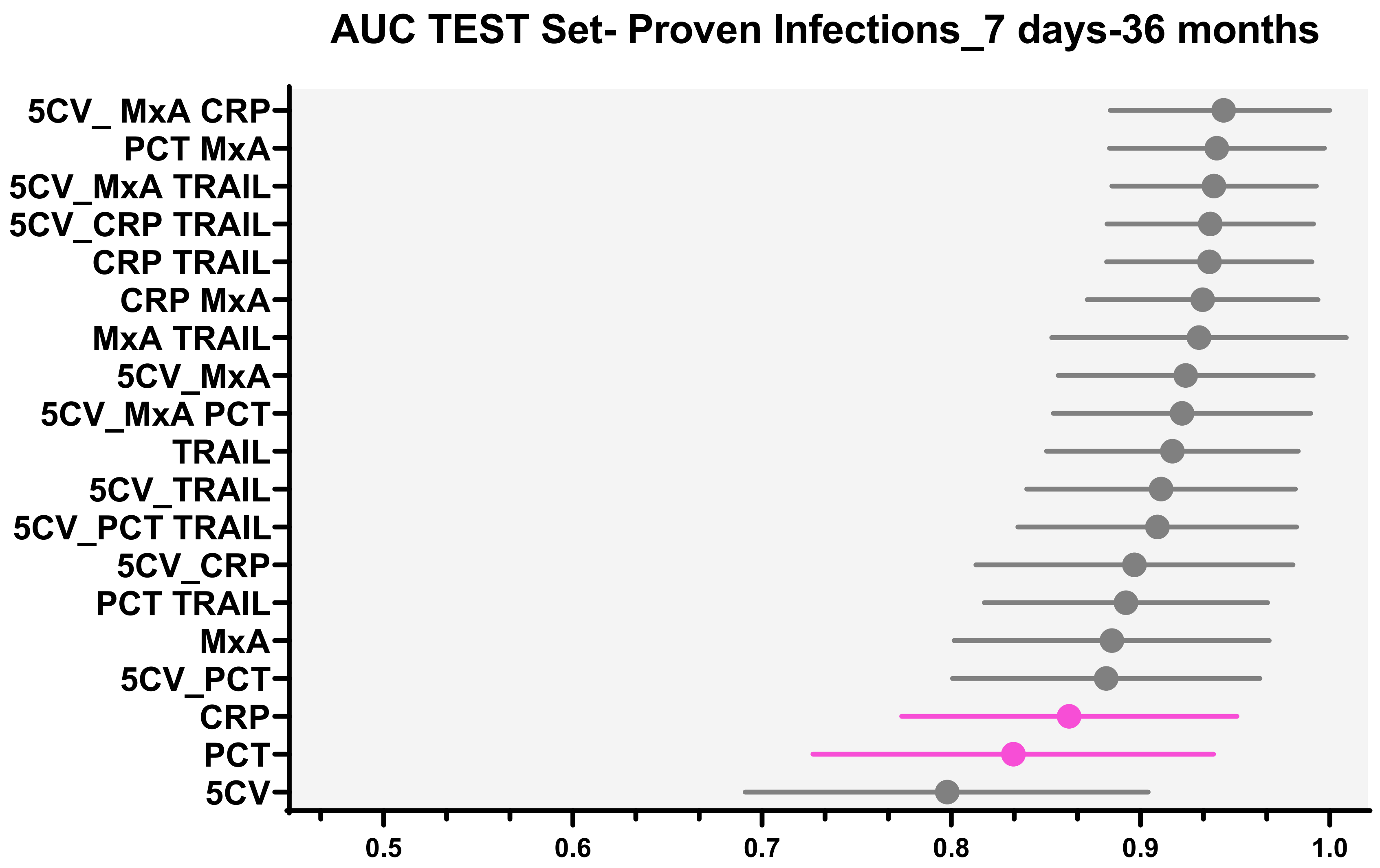

(b)

Global population (7 days – 36 months; n=64)								
	AUC	[95% CI]	Sensitivity	Specificity	PPV	NPV	LR+	LR-
xgbLinear_PCT + MxA	0.940	[0.883-0.997]	64.9%	100.0%	100.0%	67.5%	infinite	0.351
gbm_5CV + CRP + TRAIL	0.937	[0.882-0.991]	75.7%	96.3%	96.6%	74.3%	20.432	0.253
gam_5CV + MxA + TRAIL	0.939	[0.884-0.993]	73.0%	96.3%	96.4%	72.2%	19.703	0.281
gam_CRP + MxA	0.933	[0.871-0.993]	70.3%	96.3%	96.3%	70.3%	18.973	0.309
lda_5CV + MxA	0.924	[0.856-0.991]	59.5%	96.3%	95.7%	63.4%	16.054	0.421
gbm_5CV + MxA + CRP	0.944	[0.883-1.000]	59.5%	96.3%	95.7%	63.4%	16.054	0.421
rf_PCT + TRAIL	0.892	[0.817-0.967]	54.1%	96.3%	95.2%	60.5%	14.595	0.477
rf_MxA + TRAIL	0.930	[0.853-1.000]	51.4%	96.3%	95.0%	59.1%	13.865	0.505
gam_5CV + CRP	0.897	[0.813-0.980]	81.1%	92.6%	93.8%	78.1%	10.946	0.204
glmnet_5CV + MxA + PCT	0.922	[0.853-0.989]	75.7%	92.6%	93.3%	73.5%	10.216	0.263
gbm_5CV + TRAIL	0.911	[0.839-0.981]	70.3%	92.6%	92.9%	69.4%	9.486	0.321
gbm_5CV + PCT + TRAIL	0.909	[0.835-0.982]	67.6%	92.6%	92.6%	67.6%	9.122	0.350
gbm_CRP + TRAIL	0.936	[0.882-0.990]	81.1%	85.2%	88.2%	76.7%	5.473	0.222
xgbLinear_TRAIL	0.917	[0.850-0.983]	75.7%	85.2%	87.5%	71.9%	5.108	0.286
bayesglm_MxA	0.884	[0.801-0.968]	73.0%	81.5%	84.4%	68.8%	3.941	0.332
gbm_CRP	0.862	[0.773-0.950]	75.7%	77.8%	82.4%	70.0%	3.405	0.313
gbm_PCT	0.832	[0.727-0.938]	83.8%	66.7%	77.5%	75.0%	2.514	0.243
glm_5CV + PCT	0.882	[0.800-0.963]	86.5%	59.3%	74.4%	76.2%	2.123	0.228
multinom_5CV	0.798	[0.691-0.904]	67.6%	66.7%	73.5%	60.0%	2.027	0.486

Models achieving the targeted performance criteria are highlighted in green (light green: LR+ ≥ 5·67 but < 8·5 and/or LR- ≤ 0·5 but > 0·3; dark green: LR+ ≥ 8·5 and LR- ≤ 0·3).Abbreviations: AUC, area under the curve; CI, confidence interval; LR-, negative likelihood ratio; LR+, positive likelihood ratio; NPV, negative predictive value; PPV, positive predictive value.

Figure S3. Performance of machine learning-based models combining biomarkers and clinical variables in the 7 days – 36 months cohort with proven bacterial and viral infections (TEST set; n=64). (**a**) Forest plots depicting AUC (plain circles) and respective 95% CI (horizontal whiskers) of best-performing models. The reference markers CRP and PCT are shown in pink. (**b**) Performances of the models shown in panel (a). Models achieving the targeted performance criteria are highlighted in green (light green: LR+ ≥ 5.67 but < 8.5 and/or LR- ≤ 0.5 but > 0.3; dark green: LR+ ≥ 8.5 and LR- ≤ 0.3). The highest AUC was observed for 5CV/CRP/MxA (AUC [95% CI] = 0.944 [0.883–1.000]) and the best model as to both AUC and LR was found for 5CV/CRP/TRAIL (AUC [95% CI] = 0.937 [0.882–0.991], LR+ = 20.43, LR- = 0.25). The difference in AUC between 5CV/CRP/TRAIL and CRP (AUC = 0.862) was not quite significant (p=0.059) while that between 5CV/CRP/TRAIL and PCT (AUC = 0.833) was significant (p=0.022).

Table S1. Identified pathogens in patients with proven infection (N=150)

	Whole cohort (N=150)	TRAIN set (N=86)	TEST set (N=64)
**BACTERIAL INFECTIONS**			
Campylobacter jejuni	1	1	0
Enterobacteriaceae	65	35	30
Escherichia coli	62	35	27
Enterobacter cloacae	1	0	1
Enterobacter faecalis	1	0	1
Klebsiella pneumonia	1	0	1
Salmonella sp.	4	1	3
Staphylococcus sp.	7	4	3
S. aureus	6	4	2
S. hominis	1	0	1
Fusobacterium	1	1	0
Haemophilus influenzae	1	1	0
Mycoplasma sp.	2	0	2
Streptococcus sp.	13	10	3
S. pneumoniae	8	6	2
S. group A	5	4	1
**VIRAL INFECTIONS**			
Adenovirus	3	1	2
Rotavirus	9	4	5
Norovirus	1	1	0
Picornavirus (incl. enterovirus and rhinovirus)	18	8	10
Herpes simplex virus (HSV1)	1	1	0
Epstein-Barr virus	4	4	0
Parvovirus B19	1	0	1
Measles virus	1	0	1
Respiratory syncytial virus	17	11	6
Influenza virus	14	8	6
**Total number of pathogens**	**163**	**91**	**72**

Table S2. Biomarker levels according to the bacterial and viral infection status, in comparison to healthy donors

		Whole Cohort		TRAIN Set		TEST Set	
	HEALTHY DONORS [2] N=3 to 182	BACTERIAL INFECTIONS N=206	VIRAL INFECTIONS N=446	BACTERIAL INFECTIONS N=123	VIRAL INFECTIONS N=289	BACTERIAL INFECTIONS N=83	VIRAL INFECTIONS N=157
**CRP**, μg/mL, median (IQR)	0.54 (0.18–3.6)	90 (37–200)	19 (5.4–59)	84 (35–194)	18 (5.3–50)	119 (40–215)	20 (5.4–70)
**PCT**, ng/mL, median (IQR)	0.035 (0.035–0.05)	1.2 (0.33–4.5)	0.24 (0.12–0.74)	1.2 (0.28–4.5)	0.24 (0.11–0.82)	1.1 (0.39–4.8)	0.2 (0.12–0.5)
**TRAIL**, pg/mL, median (IQR)	77 (67–94)	47 (35–80)	124 (74–260)	48 (36–81)	126 (69–237)	45 (35–77)	118 (79–302)
**MxA**, ng/mL, median (IQR)	2.1 (1.5–5.3)	8.7 (3.3–41)	51 (22–99)	7.7 (3.1–42)	53 (23–104)	9.7 (3.6–37)	47 (20–84)
**IP-10**, pg/mL, median (IQR)	181 (112–288)	348 (181–669)	659 (330–1197)	315 (167–591)	653 (330–1173)	458 (194–761)	682 (333–1491)
**IL-6**, pg/mL, median (IQR)	1.9 (0.97–3.6)	60 (32–149)	28 (12–59)	69 (34–169)	30 (13–61)	48 (27–113)	25 (11–55)
**NGAL** [1], ng/mL, median (IQR)	29 (16–46)	242 (129–339)	125 (71–205)	228 (119–319)	135 (74–221)	264 (171–387)	104 (64–176)

[1] Also known as Lipocalin-2 or HNL; [2] Sera from healthy pediatric donors were used to quantify CRP, PCT, TRAIL, IP-10, IL-6 and NGAL (N=178 for CRP; N=180 for NGAL; N=182 for PCT, TRAIL, IP-10 and IL-6; see figure S1) while whole blood from healthy adult volunteers was used to quantify MxA (N=3; see figure S1), as described in the Materials and Methods section. IQR, interquartile range.

Table S3. Biomarker levels according to the bacterial and viral infection status, in the global population (7 days - 36 months) and per age category

	**Whole cohort**					
	**Global Population**		**≤ 3 months**		**> 3 months**	
	BACTERIAL INFECTIONS N=206	VIRAL INFECTIONS N=446	BACTERIAL INFECTIONS N=40	VIRAL INFECTIONS N=144	BACTERIAL INFECTIONS N=166	VIRAL INFECTIONS N=302
**CRP**, µg/mL, median (IQR)	90 (37–200)	19 (5.4–59)	54 (25–135)	6.6 (1.9–19)	97 (42–234)	26 (9.1–76)
**PCT**, ng/mL, median (IQR)	1.2 (0.33–4.5)	0.24 (0.12–0.74)	0.6 (0.22–3.4)	0.13 (0.09–0.21)	1.3 (0.39–5.1)	0.34 (0.15–1.0)
**TRAIL**, pg/mL, median (IQR)	47 (35–80)	124 (74–260)	73 (42–112)	281 (126–508)	43 (34–75)	96 (60–163)
**MxA**, ng/mL, median (IQR)	8.7 (3.3–41)	51 (22–99)	3.4 (1.4–13)	52 (26–91)	12 (4–45)	50 (21–102)
**IP-10**, pg/mL, median (IQR)	348 (181–669)	659 (330–1197)	284 (160–438)	682 (329–1778)	395 (187–763)	656 (337–1097)
**IL-6**, pg/mL, median (IQR)	60 (32–149)	28 (12–59)	50 (30–122)	23 (9.7–50)	64 (32–160)	30 (13–64)
**NGAL**[1], ng/mL, median (IQR)	242 (129–339)	125 (71–205)	144 (85–281)	74 (49–127)	259 (152–354)	151 (92–241)

	**TRAIN set**					
	**Global Population**		**≤ 3 months**		**> 3 months**	
	BACTERIAL INFECTIONS N=123	VIRAL INFECTIONS N=289	BACTERIAL INFECTIONS N=25	VIRAL INFECTIONS N=82	BACTERIAL INFECTIONS N=98	VIRAL INFECTIONS N=207
**CRP**, µg/mL, median (IQR)	84 (35–194)	18 (5.3–50)	57 (32–107)	6.1 (1.5–17)	87 (36–238)	23 (8.6–68)
**PCT**, ng/mL, median (IQR)	1.2 (0.28–4.5)	0.24 (0.11–0.82)	0.67 (0.22–3.4)	0.12 (0.08–0.21)	1.2 (0.33–5.3)	0.35 (0.14–1.1)
**TRAIL**, pg/mL, median (IQR)	48 (36–81)	126 (69–237)	75 (43–104)	289 (146–489)	42 (32–78)	98 (58–162)
**MxA**, ng/mL, median (IQR)	7.7 (3.1–42)	53 (23–104)	3.4 (1.8–22)	51 (25–96)	9.3 (3.7–47)	53 (22–104)
**IP-10**, pg/mL, median (IQR)	315 (167–591)	653 (330–1173)	296 (167–465)	553 (318–1391)	330 (165–715)	667 (356–1107)
**IL-6**, pg/mL, median (IQR)	69 (34–169)	30 (13–61)	55 (34–163)	23 (8.2–49)	72 (33–173)	34 (14–66)
**NGAL**[1], ng/mL, median (IQR)	228 (119–319)	135 (74–221)	156 (94–285)	70 (48–134)	237 (121–322)	163 (103–250)

	**TEST set**					
	**Global Population**		**≤ 3 months**		**> 3 months**	
	BACTERIAL INFECTIONS N=83	VIRAL INFECTIONS N=157	BACTERIAL INFECTIONS N=15	VIRAL INFECTIONS N=62	BACTERIAL INFECTIONS N=68	VIRAL INFECTIONS N=95
**CRP**, µg/mL, median (IQR)	119 (40–215)	20 (5.4–70)	40 (13–191)	8 (3.1–20)	133 (56–231)	42 (11–95)
**PCT**, ng/mL, median (IQR)	1.1 (0.39–4.8)	0.2 (0.12–0.5)	0.46 (0.24–3.6)	0.14 (0.11–0.24)	1.4 (0.51–5)	0.33 (0.17–0.8)
**TRAIL**, pg/mL, median (IQR)	45 (35–77)	118 (79–302)	57 (40–119)	275 (112–563)	44 (35–73)	92 (71–165)
**MxA**, ng/mL, median (IQR)	9.7 (3.6–37)	47 (20–84)	3.2 (0.77–5.7)	53 (27–84)	13 (4.9–41)	42 (15–85)
**IP-10**, pg/mL, median (IQR)	458 (194–761)	682 (333–1491)	199 (154–432)	868 (389–2070)	532 (225–930) [2]	610 (295–1041) [2]
**IL-6**, pg/mL, median (IQR)	48 (27–113)	25 (11–55)	38 (20–62) [2]	25 (11–51) [2]	50 (28–136)	25 (13–56)
**NGAL**[1], ng/mL, median (IQR)	264 (171–387)	104 (64–176)	138 (62–281)	75 (53–116)	279 (198–395)	138 (78–217)

[1] Also known as Lipocalin-2 or HNL; [2] Differences in distribution of protein concentrations between bacterial and viral infections were statistically significant ($p < 0.05$) in all cases, except for IL-6 in the ≤ 3-month group of the TEST set and for IP-10 in the > 3-month group of the TEST set ($p > 0.05$). IQR, interquartile range.
