# Supplementary material for: Performance Evaluation of Host Biomarker Combinations for the Diagnosis of Serious Bacterial Infection in Young Febrile Children: A Double-Blind, Multicentre, Observational Study
Source: J Clin Med. 2022 Nov 4;11(21):6563. doi: 10.3390/jcm11216563 (PMC9657860; doi:10.3390/jcm11216563)
Supplement: Supplementary file 1 [file jcm-11-06563-s001.zip › Supplementary Materials/Table S4.pdf]

Table S4. Performances of marker combinations using logistic regression (TRAIN set; n=412)

Global population (7 days – 36 months)							
**Models (N=98; LR- = 0.3 and LR+ = maximum)**	**AUC [95% CI]**	**Sensitivity**	**Specificity**	**PPV**	**NPV**	**LR+ [95% CI]**	**LR- [95% CI]**
Model_74: logMXA logTRAIL CRP logIL6	0.844 [0.803 ; 0.886]	77%	79%	61%	89%	3.62 [2.88 ; 4.71]	0.30 [0.21 ; 0.41]
Model_37: logIL6 logTRAIL logMXA	0.830 [0.786 ; 0.873]	77%	79%	60%	89%	3.57 [2.84 ; 4.62]	0.30 [0.21 ; 0.41]
Model_52: CRP logMXA logTRAIL	0.842 [0.800 ; 0.883]	77%	79%	60%	89%	3.57 [2.76 ; 4.50]	0.30 [0.22 ; 0.43]
Model_76: logMXA logTRAIL CRP NGAL	0.842 [0.800 ; 0.883]	77%	79%	60%	89%	3.57 [2.76 ; 4.50]	0.30 [0.22 ; 0.43]
Model_78: logMXA logTRAIL logIL6 logIP_10	0.829 [0.786 ; 0.873]	77%	79%	60%	89%	3.57 [2.84 ; 4.62]	0.30 [0.21 ; 0.41]
Model_65: logMXA logPCT logTRAIL logIL6	0.831 [0.787 ; 0.875]	77%	78%	60%	89%	3.54 [2.81 ; 4.59]	0.30 [0.21 ; 0.42]
Model_66: logMXA logPCT logTRAIL CRP	0.841 [0.799 ; 0.883]	77%	78%	60%	89%	3.54 [2.81 ; 4.59]	0.30 [0.21 ; 0.42]
Model_75: logMXA logTRAIL CRP logIP_10	0.842 [0.801 ; 0.883]	77%	78%	60%	89%	3.45 [2.69 ; 4.34]	0.30 [0.23 ; 0.43]
Model_57: NGAL logTRAIL logMXA	0.826 [0.783 ; 0.869]	77%	76%	58%	89%	3.19 [2.58 ; 4.07]	0.30 [0.21 ; 0.42]
Model_77: logMXA logTRAIL logIL6 NGAL	0.832 [0.788 ; 0.875]	77%	76%	58%	89%	3.19 [2.58 ; 4.07]	0.30 [0.21 ; 0.42]
Model_63: logMXA logPCT logTRAIL	0.830 [0.787 ; 0.874]	77%	76%	57%	89%	3.16 [2.49 ; 3.93]	0.30 [0.22 ; 0.43]
Model_64: logMXA logPCT logTRAIL logIP_10	0.831 [0.787 ; 0.874]	77%	76%	57%	89%	3.16 [2.55 ; 4.03]	0.30 [0.21 ; 0.42]
Model_67: logMXA logPCT logTRAIL NGAL	0.830 [0.786 ; 0.873]	78%	73%	55%	89%	2.90 [2.37 ; 3.65]	0.30 [0.21 ; 0.42]
Model_80: logMXA CRP NGAL logIP_10	0.821 [0.774 ; 0.869]	78%	73%	55%	89%	2.86 [2.34 ; 3.58]	0.30 [0.21 ; 0.42]
Model_69: logMXA logPCT logIP_10 logIL6	0.819 [0.770 ; 0.867]	78%	72%	55%	89%	2.83 [2.26 ; 3.46]	0.30 [0.22 ; 0.44]
Model_22: logMXA logTRAIL	0.820 [0.776 ; 0.864]	78%	72%	55%	89%	2.82 [2.26 ; 3.45]	0.30 [0.22 ; 0.44]
Model_92: logPCT logIP_10 logTRAIL logIL6	0.813 [0.768 ; 0.858]	79%	71%	54%	89%	2.71 [2.19 ; 3.29]	0.30 [0.22 ; 0.44]
Model_79: logMXA logTRAIL NGAL logIP_10	0.825 [0.782 ; 0.868]	79%	71%	53%	89%	2.69 [2.22 ; 3.33]	0.30 [0.21 ; 0.42]
Model_85: logPCT NGAL logIP_10 logTRAIL	0.807 [0.762 ; 0.852]	79%	71%	53%	89%	2.68 [2.21 ; 3.33]	0.30 [0.21 ; 0.42]
Model_68: logMXA logPCT logIP_10 NGAL	0.819 [0.771 ; 0.867]	79%	70%	53%	89%	2.65 [2.14 ; 3.21]	0.30 [0.22 ; 0.44]
Model_62: logIP_10 logPCT logTRAIL	0.805 [0.759 ; 0.850]	79%	70%	53%	89%	2.62 [2.12 ; 3.17]	0.30 [0.22 ; 0.44]
Model_94: NGAL CRP logTRAIL logIL6	0.822 [0.780 ; 0.864]	79%	69%	52%	89%	2.56 [2.08 ; 3.09]	0.31 [0.23 ; 0.45]
Model_29: logIL6 CRP logTRAIL	0.822 [0.780 ; 0.863]	80%	69%	52%	89%	2.53 [2.11 ; 3.10]	0.30 [0.21 ; 0.42]
Model_93: logPCT CRP logTRAIL logIL6	0.818 [0.776 ; 0.861]	80%	68%	52%	89%	2.53 [2.06 ; 3.04]	0.30 [0.22 ; 0.44]
Model_98: CRP logTRAIL logIL6 logIP_10	0.826 [0.783 ; 0.869]	80%	68%	51%	89%	2.48 [2.07 ; 3.03]	0.30 [0.21 ; 0.43]
Model_90: logPCT logIP_10 CRP logTRAIL	0.821 [0.777 ; 0.865]	80%	68%	51%	89%	2.47 [2.02 ; 2.96]	0.30 [0.22 ; 0.44]
Model_1: CRP	0.780 [0.731 ; 0.829]	80%	68%	51%	89%	2.45 [2.01 ; 2.93]	0.30 [0.22 ; 0.44]
Model_23: logIP_10 logTRAIL	0.785 [0.738 ; 0.831]	81%	65%	50%	89%	2.30 [1.94 ; 2.79]	0.30 [0.21 ; 0.43]
Model_45: CRP NGAL logTRAIL	0.815 [0.773 ; 0.857]	81%	65%	50%	89%	2.30 [1.90 ; 2.73]	0.30 [0.22 ; 0.45]
Model_97: NGAL logTRAIL logIL6 logIP_10	0.809 [0.764 ; 0.853]	81%	65%	50%	89%	2.30 [1.94 ; 2.79]	0.30 [0.21 ; 0.43]
Model_86: logPCT NGAL logIP_10 CRP	0.810 [0.763 ; 0.857]	81%	65%	49%	89%	2.29 [1.89 ; 2.72]	0.30 [0.22 ; 0.45]
Model_27: logTRAIL CRP	0.815 [0.774 ; 0.857]	81%	65%	49%	89%	2.28 [1.89 ; 2.70]	0.30 [0.22 ; 0.45]
Model_70: logMXA logPCT logIP_10 CRP	0.826 [0.779 ; 0.873]	81%	65%	49%	89%	2.28 [1.92 ; 2.76]	0.30 [0.20 ; 0.43]
Model_58: NGAL logTRAIL logPCT	0.791 [0.746 ; 0.837]	81%	65%	49%	89%	2.27 [1.87 ; 2.69]	0.30 [0.22 ; 0.45]
Model_47: CRP NGAL logMXA	0.818 [0.771 ; 0.865]	81%	64%	49%	89%	2.26 [1.91 ; 2.73]	0.30 [0.20 ; 0.44]
Model_51: CRP logMXA logIP_10	0.819 [0.771 ; 0.867]	81%	64%	49%	89%	2.26 [1.91 ; 2.73]	0.30 [0.20 ; 0.44]
Model_61: logIP_10 logMXA logTRAIL	0.822 [0.779 ; 0.866]	81%	64%	49%	89%	2.26 [1.91 ; 2.73]	0.30 [0.20 ; 0.44]
Model_11: logPCT logTRAIL	0.792 [0.746 ; 0.837]	81%	64%	49%	89%	2.25 [1.86 ; 2.66]	0.30 [0.22 ; 0.45]
Model_49: CRP logPCT logTRAIL	0.814 [0.772 ; 0.856]	81%	64%	49%	89%	2.25 [1.86 ; 2.66]	0.30 [0.22 ; 0.45]
Model_89: logPCT NGAL CRP logTRAIL	0.814 [0.772 ; 0.856]	81%	64%	49%	89%	2.25 [1.86 ; 2.66]	0.30 [0.22 ; 0.45]
Model_12: logPCT logIP_10	0.785 [0.736 ; 0.834]	81%	63%	48%	89%	2.18 [1.85 ; 2.63]	0.30 [0.20 ; 0.43]
Model_20: logMXA CRP	0.815 [0.767 ; 0.863]	82%	63%	48%	89%	2.18 [1.82 ; 2.57]	0.30 [0.21 ; 0.45]
Model_34: logIL6 logTRAIL logIP_10	0.805 [0.760 ; 0.851]	81%	63%	48%	89%	2.18 [1.85 ; 2.61]	0.30 [0.20 ; 0.43]
Model_95: NGAL CRP logTRAIL logIP_10	0.819 [0.776 ; 0.862]	81%	63%	48%	89%	2.18 [1.85 ; 2.61]	0.30 [0.20 ; 0.43]
Model_50: CRP logPCT logIP_10	0.808 [0.760 ; 0.855]	81%	62%	48%	89%	2.16 [1.80 ; 2.55]	0.30 [0.22 ; 0.45]
Model_54: NGAL logIP_10 logTRAIL	0.792 [0.747 ; 0.837]	81%	62%	48%	89%	2.16 [1.80 ; 2.53]	0.30 [0.22 ; 0.45]
Model_60: logIP_10 logMXA logPCT	0.815 [0.767 ; 0.864]	82%	62%	48%	89%	2.13 [1.81 ; 2.55]	0.30 [0.20 ; 0.44]
Model_72: logMXA logPCT NGAL logIL6	0.809 [0.760 ; 0.857]	82%	62%	48%	89%	2.13 [1.81 ; 2.55]	0.30 [0.20 ; 0.44]
Model_82: logMXA CRP logIP_10 logIL6	0.826 [0.779 ; 0.873]	82%	62%	48%	89%	2.12 [1.81 ; 2.54]	0.30 [0.20 ; 0.44]
Model_35: logIL6 logTRAIL logPCT	0.800 [0.755 ; 0.845]	82%	61%	47%	89%	2.09 [1.75 ; 2.44]	0.30 [0.21 ; 0.45]
Model_26: logTRAIL logIL6	0.800 [0.755 ; 0.845]	82%	61%	47%	89%	2.08 [1.78 ; 2.48]	0.30 [0.20 ; 0.43]
Model_53: CRP logIP_10 logTRAIL	0.819 [0.776 ; 0.862]	82%	61%	47%	89%	2.08 [1.75 ; 2.43]	0.30 [0.21 ; 0.45]
Model_39: logIL6 logIP_10 logPCT	0.799 [0.750 ; 0.847]	82%	60%	46%	89%	2.03 [1.74 ; 2.42]	0.30 [0.20 ; 0.44]
Model_55: NGAL logIP_10 logPCT	0.793 [0.745 ; 0.841]	82%	60%	46%	89%	2.03 [1.74 ; 2.42]	0.30 [0.20 ; 0.44]
Model_88: logPCT NGAL logIL6 logTRAIL	0.799 [0.754 ; 0.844]	82%	60%	46%	89%	2.03 [1.74 ; 2.42]	0.30 [0.20 ; 0.44]
Model_91: logPCT logIP_10 CRP logIL6	0.813 [0.766 ; 0.860]	82%	60%	46%	89%	2.03 [1.74 ; 2.42]	0.30 [0.20 ; 0.44]
Model_36: logIL6 logTRAIL NGAL	0.800 [0.755 ; 0.845]	82%	60%	46%	89%	2.03 [1.74 ; 2.41]	0.30 [0.20 ; 0.44]
Model_46: CRP NGAL logIP_10	0.795 [0.747 ; 0.843]	82%	60%	46%	89%	2.03 [1.71 ; 2.36]	0.30 [0.21 ; 0.46]
Model_73: logMXA logPCT CRP logIL6	0.821 [0.775 ; 0.868]	82%	59%	46%	89%	2.00 [1.68 ; 2.33]	0.30 [0.21 ; 0.46]
Model_17: NGAL logTRAIL	0.786 [0.741 ; 0.832]	83%	57%	45%	89%	1.93 [1.67 ; 2.28]	0.30 [0.20 ; 0.44]
Model_96: NGAL CRP logIL6 logIP_10	0.810 [0.763 ; 0.857]	83%	57%	45%	89%	1.93 [1.64 ; 2.24]	0.30 [0.21 ; 0.46]
Model_15: NGAL CRP	0.778 [0.730 ; 0.826]	83%	57%	45%	89%	1.92 [1.62 ; 2.22]	0.30 [0.21 ; 0.47]
Model_81: logMXA CRP NGAL logIL6	0.821 [0.774 ; 0.868]	83%	56%	45%	89%	1.91 [1.65 ; 2.25]	0.30 [0.20 ; 0.45]
Model_32: logIL6 CRP logMXA	0.820 [0.773 ; 0.867]	83%	56%	45%	89%	1.89 [1.64 ; 2.23]	0.30 [0.20 ; 0.45]
Model_13: logPCT logMXA	0.804 [0.755 ; 0.853]	83%	56%	45%	89%	1.89 [1.60 ; 2.19]	0.30 [0.21 ; 0.47]
Model_48: CRP logPCT logMXA	0.819 [0.772 ; 0.866]	83%	56%	45%	89%	1.89 [1.63 ; 2.23]	0.30 [0.20 ; 0.45]
Model_71: logMXA logPCT NGAL CRP	0.820 [0.773 ; 0.867]	83%	56%	45%	89%	1.89 [1.63 ; 2.23]	0.30 [0.20 ; 0.45]
Model_33: logIL6 CRP logIP_10	0.808 [0.760 ; 0.855]	83%	56%	45%	89%	1.89 [1.60 ; 2.18]	0.31 [0.22 ; 0.47]
Model_31: logIL6 CRP NGAL	0.783 [0.734 ; 0.832]	84%	54%	44%	89%	1.83 [1.59 ; 2.14]	0.30 [0.20 ; 0.45]
Model_3: logTRAIL	0.785 [0.739 ; 0.831]	84%	54%	44%	89%	1.83 [1.56 ; 2.11]	0.30 [0.21 ; 0.47]
Model_40: logIL6 logIP_10 NGAL	0.782 [0.733 ; 0.831]	84%	54%	44%	89%	1.83 [1.59 ; 2.14]	0.30 [0.20 ; 0.45]
Model_44: CRP NGAL logPCT	0.774 [0.726 ; 0.822]	84%	54%	44%	89%	1.83 [1.59 ; 2.14]	0.30 [0.20 ; 0.45]
Model_87: logPCT NGAL logIL6 CRP	0.780 [0.731 ; 0.829]	84%	54%	44%	89%	1.83 [1.56 ; 2.11]	0.30 [0.21 ; 0.47]
Model_30: logIL6 CRP logPCT	0.780 [0.731 ; 0.829]	84%	54%	44%	89%	1.82 [1.55 ; 2.09]	0.30 [0.21 ; 0.47]
Model_21: logMXA logIL6	0.792 [0.742 ; 0.843]	84%	53%	43%	89%	1.80 [1.57 ; 2.10]	0.30 [0.19 ; 0.46]
Model_42: logIL6 logMXA logPCT	0.806 [0.757 ; 0.855]	84%	53%	43%	89%	1.80 [1.56 ; 2.10]	0.30 [0.19 ; 0.46]
Model_59: NGAL logMXA logPCT	0.807 [0.758 ; 0.855]	84%	53%	43%	89%	1.80 [1.56 ; 2.10]	0.30 [0.19 ; 0.46]
Model_28: logIL6 CRP	0.782 [0.733 ; 0.831]	85%	52%	43%	89%	1.75 [1.53 ; 2.03]	0.30 [0.19 ; 0.46]
Model_9: logPCT CRP	0.775 [0.727 ; 0.823]	85%	51%	43%	89%	1.74 [1.52 ; 2.02]	0.30 [0.19 ; 0.46]
Model_25: logIP_10 logIL6	0.769 [0.719 ; 0.820]	85%	49%	42%	89%	1.67 [1.47 ; 1.92]	0.30 [0.19 ; 0.46]
Model_16: NGAL logIL6	0.726 [0.673 ; 0.778]	89%	35%	37%	89%	1.38 [1.25 ; 1.54]	0.30 [0.17 ; 0.51]
Model_2: logIL6	0.714 [0.660 ; 0.767]	89%	35%	37%	89%	1.38 [1.25 ; 1.54]	0.30 [0.17 ; 0.51]
Model_43: logIL6 NGAL logPCT	0.736 [0.684 ; 0.789]	89%	35%	37%	89%	1.38 [1.25 ; 1.54]	0.30 [0.17 ; 0.51]
Model_8: logPCT NGAL	0.718 [0.664 ; 0.771]	90%	32%	36%	89%	1.33 [1.19 ; 1.45]	0.31 [0.19 ; 0.57]
Model_19: logMXA logIP_10	0.765 [0.711 ; 0.819]	91%	31%	36%	89%	1.31 [1.20 ; 1.45]	0.30 [0.16 ; 0.53]
Model_38: logIL6 logIP_10 logMXA	0.800 [0.750 ; 0.850]	91%	31%	36%	89%	1.31 [1.20 ; 1.45]	0.30 [0.16 ; 0.53]
Model_5: logMXA	0.758 [0.703 ; 0.812]	91%	31%	36%	89%	1.31 [1.20 ; 1.45]	0.30 [0.16 ; 0.53]
Model_83: logMXA NGAL logIP_10 logIL6	0.807 [0.758 ; 0.856]	91%	31%	36%	89%	1.31 [1.18 ; 1.43]	0.30 [0.19 ; 0.58]
Model_41: logIL6 logMXA NGAL	0.799 [0.749 ; 0.848]	92%	28%	35%	89%	1.27 [1.17 ; 1.40]	0.30 [0.16 ; 0.55]
Model_56: NGAL logIP_10 logMXA	0.788 [0.736 ; 0.840]	92%	28%	35%	89%	1.27 [1.17 ; 1.40]	0.30 [0.16 ; 0.55]
Model_6: NGAL	0.673 [0.617 ; 0.729]	92%	27%	35%	89%	1.26 [1.16 ; 1.38]	0.30 [0.16 ; 0.55]
Model_14: NGAL logMXA	0.782 [0.730 ; 0.834]	92%	25%	35%	89%	1.24 [1.14 ; 1.35]	0.30 [0.15 ; 0.57]
Model_10: logPCT logIL6	0.731 [0.678 ; 0.784]	93%	25%	34%	89%	1.23 [1.13 ; 1.34]	0.30 [0.15 ; 0.57]
Model_84: logPCT NGAL logIP_10 logIL6	0.803 [0.755 ; 0.851]	95%	16%	33%	89%	1.14 [1.07 ; 1.22]	0.30 [0.13 ; 0.67]
Model_18: NGAL logIP_10	0.740 [0.688 ; 0.792]	95%	16%	33%	89%	1.14 [1.07 ; 1.22]	0.30 [0.13 ; 0.67]
Model_24: logIP_10 CRP	0.786 [0.737 ; 0.835]	95%	16%	33%	89%	1.14 [1.07 ; 1.22]	0.30 [0.13 ; 0.67]
Model_7: logPCT	0.706 [0.651 ; 0.761]	97%	11%	32%	89%	1.09 [1.06 ; 1.20]	0.30 [0.11 ; 0.67]
Model_4: logIP_10	0.678 [0.621 ; 0.735]	99%	3%	30%	89%	1.02 [1.00 ; 1.05]	0.29 [0.03 ; 2.04]

No models reached the targeted performance criteria. The best performing model in the TEST set (model 57) is highlighted in red.

Abbreviations: AUC, area under the curve; CI, confidence interval; LR-, negative likelihood ratio; LR+, positive likelihood ratio; NPV, negative predictive value; PPV, positive predictive value.
