# Supplementary material for: Performance Evaluation of Host Biomarker Combinations for the Diagnosis of Serious Bacterial Infection in Young Febrile Children: A Double-Blind, Multicentre, Observational Study
Source: J Clin Med. 2022 Nov 4;11(21):6563. doi: 10.3390/jcm11216563 (PMC9657860; doi:10.3390/jcm11216563)
Supplement: Supplementary file 1 [file jcm-11-06563-s001.zip › Supplementary Materials/Table S5.pdf]

Table S5. Performances of marker combinations using logistic regression (TEST set; parameters estimated at the threshold of the TRAIN cohort)

	Global population (7 days – 36 months; n=240)							TRAIN Cohort
Models (N=98)	AUC [95% CI]	Sensitivity	Specificity	PPV	NPV	LR+	LR-	AUC [95% CI]
Model_57: NGAL logTRAIL logMXA	0.831 [0.777 ; 0.886]	76%	82%	69%	87%	4.21	0.3	0.826 [0.783 ; 0.869]
Model_65: logMXA logPCT logTRAIL logIL6	0.828 [0.772 ; 0.883]	74%	79%	66%	85%	3.58	0.32	0.831 [0.787 ; 0.875]
Model_76: logMXA logTRAIL CRP NGAL	0.827 [0.771 ; 0.883]	76%	78%	65%	86%	3.47	0.31	0.842 [0.800 ; 0.883]
Model_66: logMXA logPCT logTRAIL CRP	0.829 [0.773 ; 0.884]	76%	78%	65%	86%	3.43	0.31	0.841 [0.799 ; 0.883]
Model_78: logMXA logTRAIL logIL6 logIP_10	0.810 [0.753 ; 0.867]	73%	78%	64%	85%	3.36	0.34	0.829 [0.786 ; 0.873]
Model_37: logIL6 logTRAIL logMXA	0.810 [0.752 ; 0.867]	73%	78%	64%	85%	3.36	0.34	0.830 [0.786 ; 0.873]
Model_64: logMXA logPCT logTRAIL logIP_10	0.832 [0.777 ; 0.887]	76%	77%	64%	86%	3.33	0.32	0.831 [0.787 ; 0.874]
Model_74: logMXA logTRAIL CRP logIL6	0.818 [0.761 ; 0.875]	74%	78%	64%	85%	3.32	0.33	0.844 [0.803 ; 0.886]
Model_63: logMXA logPCT logTRAIL	0.834 [0.778 ; 0.889]	77%	77%	64%	86%	3.29	0.3	0.830 [0.787 ; 0.874]
Model_75: logMXA logTRAIL CRP logIP_10	0.824 [0.768 ; 0.880]	76%	77%	63%	86%	3.28	0.32	0.842 [0.801 ; 0.883]
Model_52: CRP logMXA logTRAIL	0.824 [0.767 ; 0.880]	76%	77%	63%	86%	3.28	0.32	0.842 [0.800 ; 0.883]
Model_77: logMXA logTRAIL logIL6 NGAL	0.820 [0.764 ; 0.876]	74%	76%	62%	85%	3.05	0.34	0.832 [0.788 ; 0.875]
Model_67: logMXA logPCT logTRAIL NGAL	0.839 [0.784 ; 0.893]	77%	75%	62%	86%	3.03	0.31	0.830 [0.786 ; 0.873]
Model_85: logPCT NGAL logIP_10 logTRAIL	0.823 [0.768 ; 0.879]	76%	74%	61%	85%	2.87	0.33	0.807 [0.762 ; 0.852]
Model_69: logMXA logPCT logIP_10 logIL6	0.799 [0.738 ; 0.860]	67%	77%	60%	81%	2.87	0.43	0.819 [0.770 ; 0.867]
Model_22: logMXA logTRAIL	0.814 [0.757 ; 0.871]	77%	73%	60%	86%	2.85	0.32	0.820 [0.776 ; 0.864]
Model_79: logMXA logTRAIL NGAL logIP_10	0.832 [0.777 ; 0.887]	78%	72%	59%	86%	2.77	0.31	0.825 [0.782 ; 0.868]
Model_92: logPCT logIP_10 logTRAIL logIL6	0.807 [0.749 ; 0.865]	74%	73%	59%	84%	2.71	0.36	0.813 [0.768 ; 0.858]
Model_54: NGAL logIP_10 logTRAIL	0.832 [0.778 ; 0.887]	84%	69%	59%	89%	2.7	0.23	0.792 [0.747 ; 0.837]
Model_62: logIP_10 logPCT logTRAIL	0.814 [0.758 ; 0.871]	72%	73%	59%	83%	2.67	0.38	0.805 [0.759 ; 0.850]
Model_68: logMXA logPCT logIP_10 NGAL	0.821 [0.763 ; 0.878]	74%	71%	58%	84%	2.6	0.36	0.819 [0.771 ; 0.867]
Model_97: NGAL logTRAIL logIL6 logIP_10	0.805 [0.747 ; 0.862]	80%	69%	58%	87%	2.6	0.3	0.809 [0.764 ; 0.853]
Model_80: logMXA CRP NGAL logIP_10	0.802 [0.742 ; 0.862]	78%	69%	57%	86%	2.54	0.32	0.821 [0.774 ; 0.869]
Model_34: logIL6 logTRAIL logIP_10	0.790 [0.731 ; 0.849]	81%	68%	57%	87%	2.54	0.28	0.805 [0.760 ; 0.851]
Model_61: logIP_10 logMXA logTRAIL	0.819 [0.763 ; 0.876]	81%	68%	57%	87%	2.51	0.29	0.822 [0.779 ; 0.866]
Model_93: logPCT CRP logTRAIL logIL6	0.812 [0.756 ; 0.869]	77%	69%	57%	85%	2.49	0.33	0.818 [0.776 ; 0.861]
Model_45: CRP NGAL logTRAIL	0.824 [0.770 ; 0.879]	82%	67%	57%	88%	2.47	0.27	0.815 [0.773 ; 0.857]
Model_88: logPCT NGAL logIL6 logTRAIL	0.821 [0.765 ; 0.877]	82%	67%	57%	87%	2.44	0.27	0.799 [0.754 ; 0.844]
Model_89: logPCT NGAL CRP logTRAIL	0.825 [0.771 ; 0.880]	83%	66%	57%	88%	2.43	0.26	0.814 [0.772 ; 0.856]
Model_49: CRP logPCT logTRAIL	0.823 [0.768 ; 0.878]	83%	66%	57%	88%	2.43	0.26	0.814 [0.772 ; 0.856]
Model_36: logIL6 logTRAIL NGAL	0.813 [0.756 ; 0.870]	82%	66%	56%	87%	2.43	0.27	0.800 [0.755 ; 0.845]
Model_29: logIL6 CRP logTRAIL	0.812 [0.755 ; 0.868]	77%	68%	56%	85%	2.42	0.34	0.822 [0.780 ; 0.863]
Model_94: NGAL CRP logTRAIL logIL6	0.811 [0.754 ; 0.867]	77%	68%	56%	85%	2.42	0.34	0.822 [0.780 ; 0.864]
Model_90: logPCT logIP_10 CRP logTRAIL	0.811 [0.755 ; 0.867]	71%	70%	56%	82%	2.4	0.41	0.821 [0.777 ; 0.865]
Model_26: logTRAIL logIL6	0.803 [0.745 ; 0.861]	81%	66%	56%	87%	2.39	0.29	0.800 [0.755 ; 0.845]
Model_58: NGAL logTRAIL logPCT	0.837 [0.783 ; 0.890]	80%	67%	56%	86%	2.37	0.31	0.791 [0.746 ; 0.837]
Model_27: logTRAIL CRP	0.821 [0.766 ; 0.876]	83%	65%	56%	88%	2.37	0.26	0.815 [0.774 ; 0.857]
Model_35: logIL6 logTRAIL logPCT	0.815 [0.758 ; 0.872]	80%	67%	56%	86%	2.37	0.31	0.800 [0.755 ; 0.845]
Model_86: logPCT NGAL logIP_10 CRP	0.794 [0.734 ; 0.854]	75%	68%	56%	84%	2.36	0.37	0.810 [0.763 ; 0.857]
Model_17: NGAL logTRAIL	0.835 [0.781 ; 0.890]	87%	62%	55%	90%	2.31	0.21	0.786 [0.741 ; 0.832]
Model_98: CRP logTRAIL logIL6 logIP_10	0.799 [0.742 ; 0.857]	74%	68%	55%	83%	2.31	0.39	0.826 [0.783 ; 0.869]
Model_12: logPCT logIP_10	0.786 [0.724 ; 0.849]	76%	67%	55%	84%	2.31	0.36	0.785 [0.736 ; 0.834]
Model_95: NGAL CRP logTRAIL logIP_10	0.817 [0.761 ; 0.872]	81%	65%	55%	86%	2.3	0.3	0.819 [0.776 ; 0.862]
Model_23: logIP_10 logTRAIL	0.810 [0.753 ; 0.867]	84%	63%	55%	88%	2.28	0.25	0.785 [0.738 ; 0.831]
Model_55: NGAL logIP_10 logPCT	0.805 [0.745 ; 0.865]	76%	67%	55%	84%	2.26	0.36	0.793 [0.745 ; 0.841]
Model_70: logMXA logPCT logIP_10 CRP	0.806 [0.747 ; 0.866]	79%	64%	54%	85%	2.22	0.32	0.826 [0.779 ; 0.873]
Model_1: CRP	0.779 [0.717 ; 0.841]	82%	63%	54%	87%	2.22	0.29	0.780 [0.731 ; 0.829]
Model_60: logIP_10 logMXA logPCT	0.805 [0.745 ; 0.865]	81%	64%	54%	86%	2.21	0.31	0.815 [0.767 ; 0.864]
Model_50: CRP logPCT logIP_10	0.786 [0.725 ; 0.847]	75%	66%	54%	83%	2.19	0.38	0.808 [0.760 ; 0.855]
Model_39: logIL6 logIP_10 logPCT	0.781 [0.717 ; 0.845]	76%	65%	54%	84%	2.18	0.37	0.799 [0.750 ; 0.847]
Model_11: logPCT logTRAIL	0.829 [0.774 ; 0.883]	78%	64%	54%	85%	2.17	0.34	0.792 [0.746 ; 0.837]
Model_53: CRP logIP_10 logTRAIL	0.810 [0.754 ; 0.866]	84%	61%	53%	88%	2.14	0.26	0.819 [0.776 ; 0.862]
Model_15: NGAL CRP	0.797 [0.737 ; 0.857]	84%	61%	53%	88%	2.14	0.26	0.778 [0.730 ; 0.826]
Model_51: CRP logMXA logIP_10	0.783 [0.719 ; 0.848]	79%	63%	53%	85%	2.13	0.33	0.819 [0.771 ; 0.867]
Model_72: logMXA logPCT NGAL logIL6	0.801 [0.738 ; 0.863]	82%	61%	53%	86%	2.1	0.3	0.809 [0.760 ; 0.857]
Model_91: logPCT logIP_10 CRP logIL6	0.780 [0.718 ; 0.842]	75%	63%	52%	82%	2.03	0.4	0.813 [0.766 ; 0.860]
Model_47: CRP NGAL logMXA	0.791 [0.727 ; 0.855]	79%	60%	51%	85%	1.99	0.34	0.818 [0.771 ; 0.865]
Model_82: logMXA CRP logIP_10 logIL6	0.783 [0.720 ; 0.845]	79%	60%	51%	85%	1.96	0.35	0.826 [0.779 ; 0.873]
Model_96: NGAL CRP logIL6 logIP_10	0.772 [0.710 ; 0.834]	72%	63%	51%	81%	1.96	0.44	0.810 [0.763 ; 0.857]
Model_9: logPCT CRP	0.794 [0.733 ; 0.854]	88%	55%	51%	90%	1.95	0.22	0.775 [0.727 ; 0.823]
Model_20: logMXA CRP	0.775 [0.707 ; 0.842]	79%	59%	50%	84%	1.93	0.35	0.815 [0.767 ; 0.863]
Model_44: CRP NGAL logPCT	0.803 [0.743 ; 0.862]	84%	56%	50%	87%	1.9	0.28	0.774 [0.726 ; 0.822]
Model_13: logPCT logMXA	0.791 [0.726 ; 0.857]	82%	57%	50%	85%	1.88	0.32	0.804 [0.755 ; 0.853]
Model_46: CRP NGAL logIP_10	0.785 [0.724 ; 0.845]	75%	60%	50%	82%	1.86	0.42	0.795 [0.747 ; 0.843]
Model_48: CRP logPCT logMXA	0.791 [0.726 ; 0.857]	81%	57%	50%	85%	1.85	0.35	0.819 [0.772 ; 0.866]
Model_3: logTRAIL	0.815 [0.758 ; 0.871]	87%	53%	49%	88%	1.84	0.25	0.785 [0.739 ; 0.831]
Model_59: NGAL logMXA logPCT	0.809 [0.748 ; 0.871]	85%	53%	49%	87%	1.83	0.28	0.807 [0.758 ; 0.855]
Model_40: logIL6 logIP_10 NGAL	0.762 [0.698 ; 0.826]	70%	62%	49%	80%	1.83	0.49	0.782 [0.733 ; 0.831]
Model_42: logIL6 logMXA logPCT	0.787 [0.722 ; 0.852]	82%	55%	49%	85%	1.82	0.33	0.806 [0.757 ; 0.855]
Model_73: logMXA logPCT CRP logIL6	0.787 [0.722 ; 0.852]	81%	56%	49%	84%	1.82	0.35	0.821 [0.775 ; 0.868]
Model_33: logIL6 CRP logIP_10	0.763 [0.700 ; 0.826]	75%	59%	49%	81%	1.8	0.43	0.808 [0.760 ; 0.855]
Model_71: logMXA logPCT NGAL CRP	0.798 [0.733 ; 0.862]	81%	55%	49%	84%	1.77	0.36	0.820 [0.773 ; 0.867]
Model_16: NGAL logIL6	0.743 [0.678 ; 0.809]	90%	48%	48%	90%	1.73	0.2	0.726 [0.673 ; 0.778]
Model_32: logIL6 CRP logMXA	0.773 [0.707 ; 0.840]	82%	51%	47%	84%	1.68	0.36	0.820 [0.773 ; 0.867]
Model_28: logIL6 CRP	0.758 [0.692 ; 0.824]	80%	52%	47%	83%	1.67	0.39	0.782 [0.733 ; 0.831]
Model_30: logIL6 CRP logPCT	0.768 [0.703 ; 0.832]	78%	53%	47%	82%	1.66	0.41	0.780 [0.731 ; 0.829]
Model_31: logIL6 CRP NGAL	0.764 [0.698 ; 0.829]	77%	54%	47%	82%	1.66	0.43	0.783 [0.734 ; 0.832]
Model_81: logMXA CRP NGAL logIL6	0.778 [0.712 ; 0.843]	81%	51%	47%	83%	1.65	0.38	0.821 [0.774 ; 0.868]
Model_87: logPCT NGAL logIL6 CRP	0.769 [0.705 ; 0.834]	77%	53%	47%	81%	1.64	0.43	0.780 [0.731 ; 0.829]
Model_25: logIP_10 logIL6	0.714 [0.646 ; 0.781]	72%	55%	46%	79%	1.6	0.51	0.769 [0.719 ; 0.820]
Model_43: logIL6 NGAL logPCT	0.784 [0.721 ; 0.847]	89%	44%	46%	88%	1.59	0.25	0.736 [0.684 ; 0.789]
Model_8: logPCT NGAL	0.814 [0.755 ; 0.874]	92%	41%	45%	90%	1.54	0.21	0.718 [0.664 ; 0.771]
Model_21: logMXA logIL6	0.736 [0.668 ; 0.804]	79%	48%	45%	82%	1.53	0.43	0.792 [0.742 ; 0.843]
Model_83: logMXA NGAL logIP_10 logIL6	0.784 [0.723 ; 0.846]	93%	37%	43%	91%	1.46	0.2	0.807 [0.758 ; 0.856]
Model_6: NGAL	0.799 [0.739 ; 0.859]	93%	34%	43%	90%	1.41	0.21	0.673 [0.617 ; 0.729]
Model_2: logIL6	0.676 [0.606 ; 0.747]	86%	40%	43%	84%	1.41	0.37	0.714 [0.660 ; 0.767]
Model_56: NGAL logIP_10 logMXA	0.800 [0.742 ; 0.859]	93%	34%	43%	90%	1.4	0.22	0.788 [0.736 ; 0.840]
Model_38: logIL6 logIP_10 logMXA	0.748 [0.682 ; 0.813]	90%	33%	42%	87%	1.35	0.29	0.800 [0.750 ; 0.850]
Model_84: logPCT NGAL logIP_10 logIL6	0.795 [0.733 ; 0.857]	95%	29%	42%	92%	1.34	0.17	0.803 [0.755 ; 0.851]
Model_24: logIP_10 CRP	0.763 [0.701 ; 0.826]	96%	26%	41%	93%	1.31	0.14	0.786 [0.737 ; 0.835]
Model_10: logPCT logIL6	0.767 [0.701 ; 0.832]	93%	28%	41%	88%	1.3	0.26	0.731 [0.678 ; 0.784]
Model_14: NGAL logMXA	0.791 [0.730 ; 0.853]	94%	27%	40%	89%	1.29	0.23	0.782 [0.730 ; 0.834]
Model_18: NGAL logIP_10	0.787 [0.725 ; 0.849]	95%	26%	41%	91%	1.29	0.19	0.740 [0.688 ; 0.792]
Model_41: logIL6 logMXA NGAL	0.773 [0.709 ; 0.837]	93%	28%	40%	88%	1.28	0.27	0.799 [0.749 ; 0.848]
Model_19: logMXA logIP_10	0.716 [0.645 ; 0.788]	89%	28%	40%	83%	1.24	0.39	0.765 [0.711 ; 0.819]
Model_5: logMXA	0.709 [0.637 ; 0.782]	88%	22%	37%	77%	1.12	0.56	0.758 [0.703 ; 0.812]
Model_7: logPCT	0.789 [0.725 ; 0.853]	94%	9%	36%	74%	1.03	0.67	0.706 [0.651 ; 0.761]
Model_4: logIP_10	0.643 [0.570 ; 0.716]	99%	5%	35%	88%	1.03	0.27	0.678 [0.621 ; 0.735]

≤ 3 months (7 – 91 days; n=77)							
**Models (N=98)**	**AUC [95% CI]**	**Sensitivity**	**Specificity**	**PPV**	**NPV**	**LR+**	**LR-**
Model_37: logIL6 logTRAIL logMXA	0.884 [0.780 ; 0.989]	71%	93%	71%	93%	10.89	0.31
Model_78: logMXA logTRAIL logIL6 logIP_10	0.884 [0.780 ; 0.989]	71%	93%	71%	93%	10.89	0.31
Model_75: logMXA logTRAIL CRP logIP_10	0.896 [0.775 ; 1.000]	86%	92%	71%	97%	10.46	0.16
Model_52: CRP logMXA logTRAIL	0.895 [0.771 ; 1.000]	86%	92%	71%	97%	10.46	0.16
Model_76: logMXA logTRAIL CRP NGAL	0.891 [0.764 ; 1.000]	86%	92%	71%	97%	10.46	0.16
Model_66: logMXA logPCT logTRAIL CRP	0.889 [0.764 ; 1.000]	86%	92%	71%	97%	10.29	0.16
Model_74: logMXA logTRAIL CRP logIL6	0.898 [0.791 ; 1.000]	79%	92%	69%	95%	9.59	0.23
Model_57: NGAL logTRAIL logMXA	0.883 [0.760 ; 1.000]	79%	92%	69%	95%	9.59	0.23
Model_67: logMXA logPCT logTRAIL NGAL	0.885 [0.764 ; 1.000]	79%	92%	69%	95%	9.43	0.23
Model_64: logMXA logPCT logTRAIL logIP_10	0.882 [0.761 ; 1.000]	79%	92%	69%	95%	9.43	0.23
Model_63: logMXA logPCT logTRAIL	0.881 [0.760 ; 1.000]	79%	92%	69%	95%	9.43	0.23
Model_77: logMXA logTRAIL logIL6 NGAL	0.891 [0.789 ; 0.993]	71%	92%	67%	93%	8.71	0.31
Model_65: logMXA logPCT logTRAIL logIL6	0.889 [0.787 ; 0.991]	71%	92%	67%	93%	8.57	0.31
Model_22: logMXA logTRAIL	0.878 [0.756 ; 1.000]	79%	90%	65%	95%	7.99	0.24
Model_45: CRP NGAL logTRAIL	0.860 [0.750 ; 0.971]	67%	90%	63%	92%	6.89	0.37
Model_79: logMXA logTRAIL NGAL logIP_10	0.883 [0.765 ; 1.000]	79%	89%	61%	95%	6.85	0.24
Model_54: NGAL logIP_10 logTRAIL	0.861 [0.752 ; 0.971]	73%	89%	61%	93%	6.5	0.3
Model_15: NGAL CRP	0.775 [0.615 ; 0.936]	67%	89%	59%	92%	5.91	0.38
Model_49: CRP logPCT logTRAIL	0.860 [0.751 ; 0.969]	67%	89%	59%	92%	5.81	0.38
Model_89: logPCT NGAL CRP logTRAIL	0.859 [0.750 ; 0.968]	67%	89%	59%	92%	5.81	0.38
Model_90: logPCT logIP_10 CRP logTRAIL	0.873 [0.758 ; 0.989]	73%	87%	58%	93%	5.59	0.31
Model_85: logPCT NGAL logIP_10 logTRAIL	0.858 [0.741 ; 0.974]	73%	87%	58%	93%	5.59	0.31
Model_86: logPCT NGAL logIP_10 CRP	0.864 [0.744 ; 0.985]	80%	85%	57%	95%	5.42	0.24
Model_92: logPCT logIP_10 logTRAIL logIL6	0.861 [0.756 ; 0.967]	80%	85%	57%	95%	5.42	0.24
Model_55: NGAL logIP_10 logPCT	0.854 [0.734 ; 0.973]	80%	85%	57%	95%	5.42	0.24
Model_69: logMXA logPCT logIP_10 logIL6	0.879 [0.766 ; 0.991]	71%	87%	56%	93%	5.36	0.33
Model_61: logIP_10 logMXA logTRAIL	0.888 [0.776 ; 0.999]	79%	85%	55%	95%	5.33	0.25
Model_80: logMXA CRP NGAL logIP_10	0.886 [0.756 ; 1.000]	86%	84%	55%	96%	5.23	0.17
Model_93: logPCT CRP logTRAIL logIL6	0.848 [0.740 ; 0.957]	60%	89%	56%	90%	5.23	0.45
Model_68: logMXA logPCT logIP_10 NGAL	0.882 [0.751 ; 1.000]	86%	83%	55%	96%	5.14	0.17
Model_62: logIP_10 logPCT logTRAIL	0.859 [0.744 ; 0.975]	67%	87%	56%	91%	5.08	0.38
Model_27: logTRAIL CRP	0.861 [0.751 ; 0.972]	73%	86%	55%	93%	5.05	0.31
Model_29: logIL6 CRP logTRAIL	0.848 [0.739 ; 0.957]	60%	87%	53%	90%	4.65	0.46
Model_94: NGAL CRP logTRAIL logIL6	0.846 [0.738 ; 0.955]	60%	87%	53%	90%	4.65	0.46
Model_1: CRP	0.766 [0.607 ; 0.925]	60%	87%	53%	90%	4.65	0.46
Model_95: NGAL CRP logTRAIL logIP_10	0.872 [0.761 ; 0.983]	73%	84%	52%	93%	4.55	0.32
Model_17: NGAL logTRAIL	0.855 [0.746 ; 0.964]	73%	84%	52%	93%	4.55	0.32
Model_58: NGAL logTRAIL logPCT	0.850 [0.741 ; 0.960]	67%	85%	53%	91%	4.52	0.39
Model_11: logPCT logTRAIL	0.845 [0.734 ; 0.955]	67%	85%	53%	91%	4.52	0.39
Model_12: logPCT logIP_10	0.854 [0.734 ; 0.973]	80%	82%	52%	94%	4.44	0.24
Model_98: CRP logTRAIL logIL6 logIP_10	0.853 [0.745 ; 0.960]	73%	82%	50%	93%	4.13	0.32
Model_23: logIP_10 logTRAIL	0.849 [0.740 ; 0.959]	73%	82%	50%	93%	4.13	0.32
Model_97: NGAL logTRAIL logIL6 logIP_10	0.849 [0.742 ; 0.957]	80%	81%	50%	94%	4.13	0.25
Model_88: logPCT NGAL logIL6 logTRAIL	0.839 [0.731 ; 0.947]	73%	82%	50%	93%	4.07	0.33
Model_9: logPCT CRP	0.804 [0.648 ; 0.961]	80%	80%	50%	94%	4.07	0.25
Model_51: CRP logMXA logIP_10	0.890 [0.765 ; 1.000]	86%	79%	48%	96%	4.02	0.18
Model_60: logIP_10 logMXA logPCT	0.885 [0.756 ; 1.000]	86%	78%	48%	96%	3.96	0.18
Model_53: CRP logIP_10 logTRAIL	0.872 [0.761 ; 0.983]	80%	79%	48%	94%	3.82	0.25
Model_34: logIL6 logTRAIL logIP_10	0.840 [0.733 ; 0.947]	80%	79%	48%	94%	3.82	0.25
Model_50: CRP logPCT logIP_10	0.866 [0.745 ; 0.986]	80%	79%	48%	94%	3.75	0.25
Model_91: logPCT logIP_10 CRP logIL6	0.856 [0.745 ; 0.966]	80%	79%	48%	94%	3.75	0.25
Model_44: CRP NGAL logPCT	0.799 [0.644 ; 0.954]	73%	80%	48%	93%	3.73	0.33
Model_35: logIL6 logTRAIL logPCT	0.832 [0.723 ; 0.941]	67%	82%	48%	91%	3.7	0.41
Model_70: logMXA logPCT logIP_10 CRP	0.895 [0.766 ; 1.000]	86%	77%	46%	96%	3.67	0.19
Model_36: logIL6 logTRAIL NGAL	0.833 [0.725 ; 0.941]	73%	79%	46%	93%	3.5	0.34
Model_39: logIL6 logIP_10 logPCT	0.859 [0.747 ; 0.971]	80%	77%	46%	94%	3.49	0.26
Model_3: logTRAIL	0.837 [0.723 ; 0.950]	73%	77%	44%	92%	3.25	0.34
Model_47: CRP NGAL logMXA	0.875 [0.737 ; 1.000]	86%	72%	41%	96%	3.08	0.2
Model_72: logMXA logPCT NGAL logIL6	0.880 [0.777 ; 0.982]	86%	72%	41%	96%	3.03	0.2
Model_46: CRP NGAL logIP_10	0.842 [0.719 ; 0.964]	73%	76%	42%	92%	3.03	0.35
Model_26: logTRAIL logIL6	0.826 [0.718 ; 0.934]	67%	77%	42%	91%	2.95	0.43
Model_96: NGAL CRP logIL6 logIP_10	0.822 [0.704 ; 0.939]	67%	77%	42%	91%	2.95	0.43
Model_20: logMXA CRP	0.885 [0.759 ; 1.000]	86%	71%	40%	96%	2.91	0.2
Model_73: logMXA logPCT CRP logIL6	0.882 [0.769 ; 0.995]	86%	70%	40%	96%	2.86	0.2
Model_82: logMXA CRP logIP_10 logIL6	0.883 [0.777 ; 0.989]	86%	69%	39%	96%	2.75	0.21
Model_48: CRP logPCT logMXA	0.889 [0.767 ; 1.000]	86%	68%	39%	95%	2.71	0.21
Model_71: logMXA logPCT NGAL CRP	0.887 [0.763 ; 1.000]	86%	68%	39%	95%	2.71	0.21
Model_59: NGAL logMXA logPCT	0.879 [0.759 ; 0.998]	86%	68%	39%	95%	2.71	0.21
Model_33: logIL6 CRP logIP_10	0.820 [0.700 ; 0.941]	73%	73%	39%	92%	2.68	0.37
Model_13: logPCT logMXA	0.876 [0.764 ; 0.989]	79%	70%	38%	93%	2.62	0.31
Model_40: logIL6 logIP_10 NGAL	0.805 [0.688 ; 0.922]	67%	74%	39%	90%	2.58	0.45
Model_42: logIL6 logMXA logPCT	0.871 [0.769 ; 0.974]	86%	67%	38%	95%	2.57	0.21
Model_8: logPCT NGAL	0.792 [0.640 ; 0.945]	87%	64%	37%	95%	2.4	0.21
Model_81: logMXA CRP NGAL logIL6	0.874 [0.756 ; 0.991]	86%	64%	35%	95%	2.38	0.22
Model_32: logIL6 CRP logMXA	0.876 [0.761 ; 0.991]	86%	62%	34%	95%	2.27	0.23
Model_43: logIL6 NGAL logPCT	0.764 [0.622 ; 0.906]	93%	56%	34%	97%	2.11	0.12
Model_25: logIP_10 logIL6	0.781 [0.659 ; 0.903]	80%	61%	33%	93%	2.07	0.33
Model_16: NGAL logIL6	0.683 [0.532 ; 0.834]	87%	55%	32%	94%	1.92	0.24
Model_28: logIL6 CRP	0.716 [0.555 ; 0.877]	60%	66%	30%	87%	1.77	0.61
Model_83: logMXA NGAL logIP_10 logIL6	0.879 [0.779 ; 0.980]	93%	46%	28%	97%	1.72	0.16
Model_56: NGAL logIP_10 logMXA	0.877 [0.751 ; 1.000]	93%	46%	28%	97%	1.72	0.16
Model_30: logIL6 CRP logPCT	0.750 [0.599 ; 0.901]	53%	69%	30%	86%	1.71	0.68
Model_87: logPCT NGAL logIL6 CRP	0.745 [0.594 ; 0.897]	53%	69%	30%	86%	1.71	0.68
Model_24: logIP_10 CRP	0.847 [0.724 ; 0.970]	93%	45%	29%	97%	1.7	0.15
Model_21: logMXA logIL6	0.836 [0.719 ; 0.953]	86%	49%	28%	94%	1.69	0.29
Model_84: logPCT NGAL logIP_10 logIL6	0.855 [0.743 ; 0.966]	93%	44%	29%	96%	1.68	0.15
Model_18: NGAL logIP_10	0.828 [0.707 ; 0.949]	93%	44%	29%	96%	1.65	0.15
Model_31: logIL6 CRP NGAL	0.723 [0.562 ; 0.883]	53%	68%	29%	86%	1.65	0.69
Model_38: logIL6 logIP_10 logMXA	0.856 [0.748 ; 0.964]	100%	36%	26%	100%	1.56	0
Model_6: NGAL	0.725 [0.569 ; 0.880]	73%	52%	27%	89%	1.52	0.52
Model_14: NGAL logMXA	0.865 [0.738 ; 0.992]	100%	31%	25%	100%	1.45	0
Model_10: logPCT logIL6	0.769 [0.628 ; 0.911]	93%	34%	26%	96%	1.42	0.19
Model_19: logMXA logIP_10	0.840 [0.705 ; 0.974]	100%	28%	24%	100%	1.39	0
Model_2: logIL6	0.626 [0.472 ; 0.780]	80%	42%	25%	90%	1.38	0.48
Model_41: logIL6 logMXA NGAL	0.858 [0.747 ; 0.970]	100%	25%	23%	100%	1.33	0
Model_5: logMXA	0.828 [0.685 ; 0.971]	93%	21%	21%	93%	1.18	0.34
Model_4: logIP_10	0.819 [0.696 ; 0.943]	100%	7%	21%	100%	1.07	0
Model_7: logPCT	0.792 [0.644 ; 0.941]	93%	12%	21%	88%	1.05	0.58

> 3 months (92 days – 36 months; n=163)							
**Models (N=98)**	**AUC [95% CI]**	**Sensitivity**	**Specificity**	**PPV**	**NPV**	**LR+**	**LR-**
Model_57: NGAL logTRAIL logMXA	0.782 [0.710 ; 0.854]	75%	76%	69%	81%	3.1	0.33
Model_65: logMXA logPCT logTRAIL logIL6	0.784 [0.712 ; 0.857]	75%	71%	65%	80%	2.61	0.35
Model_76: logMXA logTRAIL CRP NGAL	0.772 [0.698 ; 0.846]	74%	70%	63%	79%	2.41	0.38
Model_66: logMXA logPCT logTRAIL CRP	0.776 [0.703 ; 0.850]	74%	69%	63%	78%	2.38	0.38
Model_64: logMXA logPCT logTRAIL logIP_10	0.787 [0.715 ; 0.859]	75%	68%	63%	79%	2.35	0.37
Model_74: logMXA logTRAIL CRP logIL6	0.764 [0.690 ; 0.839]	74%	68%	63%	78%	2.33	0.39
Model_78: logMXA logTRAIL logIL6 logIP_10	0.760 [0.685 ; 0.835]	74%	68%	63%	78%	2.33	0.39
Model_37: logIL6 logTRAIL logMXA	0.759 [0.684 ; 0.834]	74%	68%	63%	78%	2.33	0.39
Model_63: logMXA logPCT logTRAIL	0.791 [0.719 ; 0.862]	77%	67%	63%	80%	2.32	0.35
Model_75: logMXA logTRAIL CRP logIP_10	0.767 [0.693 ; 0.842]	74%	67%	62%	78%	2.25	0.39
Model_52: CRP logMXA logTRAIL	0.766 [0.692 ; 0.841]	74%	67%	62%	78%	2.25	0.39
Model_69: logMXA logPCT logIP_10 logIL6	0.754 [0.677 ; 0.831]	66%	70%	62%	74%	2.22	0.48
Model_85: logPCT NGAL logIP_10 logTRAIL	0.789 [0.716 ; 0.861]	77%	65%	61%	79%	2.18	0.36
Model_77: logMXA logTRAIL logIL6 NGAL	0.774 [0.700 ; 0.847]	75%	65%	61%	79%	2.16	0.38
Model_67: logMXA logPCT logTRAIL NGAL	0.796 [0.725 ; 0.866]	77%	64%	61%	79%	2.11	0.37
Model_97: NGAL logTRAIL logIL6 logIP_10	0.770 [0.696 ; 0.844]	79%	62%	60%	81%	2.1	0.33
Model_34: logIL6 logTRAIL logIP_10	0.750 [0.673 ; 0.827]	81%	61%	60%	82%	2.08	0.31
Model_92: logPCT logIP_10 logTRAIL logIL6	0.767 [0.692 ; 0.843]	72%	65%	60%	76%	2.05	0.43
Model_26: logTRAIL logIL6	0.773 [0.698 ; 0.848]	84%	59%	59%	84%	2.04	0.27
Model_62: logIP_10 logPCT logTRAIL	0.774 [0.700 ; 0.849]	74%	64%	60%	77%	2.03	0.42
Model_22: logMXA logTRAIL	0.759 [0.684 ; 0.835]	77%	62%	59%	79%	2.02	0.38
Model_79: logMXA logTRAIL NGAL logIP_10	0.783 [0.711 ; 0.854]	78%	61%	59%	80%	2	0.36
Model_36: logIL6 logTRAIL NGAL	0.787 [0.714 ; 0.859]	84%	58%	59%	83%	1.99	0.28
Model_68: logMXA logPCT logIP_10 NGAL	0.776 [0.702 ; 0.849]	72%	64%	59%	76%	1.99	0.44
Model_54: NGAL logIP_10 logTRAIL	0.800 [0.730 ; 0.870]	87%	56%	58%	86%	1.96	0.24
Model_88: logPCT NGAL logIL6 logTRAIL	0.798 [0.727 ; 0.870]	84%	56%	58%	83%	1.92	0.29
Model_80: logMXA CRP NGAL logIP_10	0.744 [0.667 ; 0.822]	77%	60%	58%	78%	1.91	0.39
Model_35: logIL6 logTRAIL logPCT	0.791 [0.718 ; 0.863]	82%	56%	58%	82%	1.89	0.31
Model_61: logIP_10 logMXA logTRAIL	0.766 [0.692 ; 0.841]	81%	57%	57%	81%	1.87	0.34
Model_93: logPCT CRP logTRAIL logIL6	0.777 [0.704 ; 0.850]	81%	56%	57%	80%	1.85	0.34
Model_29: logIL6 CRP logTRAIL	0.776 [0.703 ; 0.849]	81%	56%	57%	80%	1.83	0.34
Model_94: NGAL CRP logTRAIL logIL6	0.775 [0.701 ; 0.848]	81%	56%	57%	80%	1.83	0.34
Model_58: NGAL logTRAIL logPCT	0.814 [0.746 ; 0.882]	82%	54%	57%	81%	1.8	0.33
Model_98: CRP logTRAIL logIL6 logIP_10	0.757 [0.682 ; 0.832]	74%	59%	56%	76%	1.79	0.45
Model_70: logMXA logPCT logIP_10 CRP	0.748 [0.671 ; 0.826]	78%	56%	56%	78%	1.79	0.39
Model_89: logPCT NGAL CRP logTRAIL	0.788 [0.717 ; 0.860]	87%	51%	56%	84%	1.77	0.26
Model_49: CRP logPCT logTRAIL	0.784 [0.713 ; 0.856]	87%	51%	56%	84%	1.77	0.26
Model_72: logMXA logPCT NGAL logIL6	0.763 [0.685 ; 0.840]	81%	54%	56%	80%	1.77	0.35
Model_45: CRP NGAL logTRAIL	0.785 [0.714 ; 0.857]	85%	52%	56%	83%	1.76	0.29
Model_27: logTRAIL CRP	0.779 [0.706 ; 0.851]	85%	52%	56%	83%	1.76	0.29
Model_12: logPCT logIP_10	0.742 [0.662 ; 0.822]	75%	57%	56%	76%	1.76	0.44
Model_39: logIL6 logIP_10 logPCT	0.741 [0.660 ; 0.822]	75%	57%	56%	76%	1.76	0.44
Model_23: logIP_10 logTRAIL	0.770 [0.695 ; 0.844]	87%	51%	56%	84%	1.75	0.26
Model_90: logPCT logIP_10 CRP logTRAIL	0.760 [0.685 ; 0.835]	71%	60%	56%	74%	1.75	0.49
Model_17: NGAL logTRAIL	0.811 [0.742 ; 0.881]	90%	48%	56%	87%	1.74	0.21
Model_95: NGAL CRP logTRAIL logIP_10	0.770 [0.696 ; 0.843]	82%	53%	55%	81%	1.74	0.34
Model_60: logIP_10 logMXA logPCT	0.757 [0.680 ; 0.833]	79%	54%	56%	79%	1.74	0.38
Model_86: logPCT NGAL logIP_10 CRP	0.744 [0.666 ; 0.823]	74%	57%	56%	75%	1.73	0.46
Model_50: CRP logPCT logIP_10	0.730 [0.650 ; 0.810]	74%	57%	56%	75%	1.73	0.46
Model_82: logMXA CRP logIP_10 logIL6	0.731 [0.652 ; 0.810]	78%	54%	55%	77%	1.68	0.41
Model_53: CRP logIP_10 logTRAIL	0.758 [0.684 ; 0.833]	85%	48%	54%	82%	1.65	0.3
Model_47: CRP NGAL logMXA	0.740 [0.660 ; 0.820]	78%	53%	54%	77%	1.65	0.42
Model_1: CRP	0.738 [0.660 ; 0.815]	87%	47%	54%	83%	1.65	0.28
Model_51: CRP logMXA logIP_10	0.726 [0.645 ; 0.807]	78%	53%	54%	77%	1.65	0.42
Model_55: NGAL logIP_10 logPCT	0.767 [0.690 ; 0.845]	75%	54%	54%	75%	1.64	0.46
Model_11: logPCT logTRAIL	0.804 [0.735 ; 0.874]	81%	50%	54%	78%	1.62	0.38
Model_20: logMXA CRP	0.722 [0.640 ; 0.805]	78%	52%	54%	77%	1.61	0.43
Model_16: NGAL logIL6	0.741 [0.664 ; 0.818]	91%	43%	53%	87%	1.6	0.2
Model_96: NGAL CRP logIL6 logIP_10	0.732 [0.653 ; 0.810]	74%	54%	53%	74%	1.59	0.49
Model_13: logPCT logMXA	0.756 [0.677 ; 0.836]	82%	48%	53%	79%	1.58	0.37
Model_91: logPCT logIP_10 CRP logIL6	0.732 [0.652 ; 0.812]	74%	53%	53%	74%	1.57	0.5
Model_48: CRP logPCT logMXA	0.742 [0.662 ; 0.823]	79%	49%	53%	77%	1.56	0.42
Model_42: logIL6 logMXA logPCT	0.751 [0.672 ; 0.830]	81%	48%	53%	78%	1.55	0.4
Model_15: NGAL CRP	0.766 [0.692 ; 0.840]	88%	42%	52%	83%	1.52	0.28
Model_40: logIL6 logIP_10 NGAL	0.735 [0.656 ; 0.814]	71%	54%	52%	72%	1.52	0.55
Model_59: NGAL logMXA logPCT	0.773 [0.696 ; 0.849]	85%	44%	52%	80%	1.51	0.34
Model_73: logMXA logPCT CRP logIL6	0.742 [0.662 ; 0.821]	79%	47%	52%	76%	1.49	0.44
Model_31: logIL6 CRP NGAL	0.743 [0.665 ; 0.821]	82%	44%	51%	78%	1.48	0.4
Model_46: CRP NGAL logIP_10	0.737 [0.660 ; 0.815]	75%	50%	52%	73%	1.48	0.51
Model_28: logIL6 CRP	0.737 [0.658 ; 0.816]	84%	43%	51%	79%	1.48	0.38
Model_33: logIL6 CRP logIP_10	0.720 [0.640 ; 0.800]	75%	50%	52%	73%	1.48	0.51
Model_21: logMXA logIL6	0.703 [0.621 ; 0.784]	78%	47%	52%	75%	1.48	0.47
Model_71: logMXA logPCT NGAL CRP	0.749 [0.669 ; 0.829]	79%	46%	51%	75%	1.46	0.45
Model_30: logIL6 CRP logPCT	0.743 [0.665 ; 0.821]	84%	43%	51%	78%	1.46	0.38
Model_9: logPCT CRP	0.754 [0.678 ; 0.830]	90%	38%	51%	84%	1.45	0.27
Model_32: logIL6 CRP logMXA	0.730 [0.649 ; 0.810]	81%	44%	51%	76%	1.45	0.43
Model_44: CRP NGAL logPCT	0.769 [0.695 ; 0.843]	87%	39%	51%	80%	1.43	0.34
Model_87: logPCT NGAL logIL6 CRP	0.745 [0.666 ; 0.823]	82%	43%	51%	77%	1.43	0.42
Model_25: logIP_10 logIL6	0.681 [0.598 ; 0.764]	71%	51%	51%	71%	1.43	0.58
Model_3: logTRAIL	0.782 [0.709 ; 0.855]	90%	37%	50%	83%	1.42	0.28
Model_81: logMXA CRP NGAL logIL6	0.734 [0.654 ; 0.814]	79%	43%	50%	75%	1.4	0.48
Model_2: logIL6	0.682 [0.599 ; 0.765]	87%	38%	50%	80%	1.4	0.35
Model_43: logIL6 NGAL logPCT	0.773 [0.699 ; 0.847]	88%	36%	50%	81%	1.38	0.33
Model_83: logMXA NGAL logIP_10 logIL6	0.739 [0.662 ; 0.817]	93%	31%	49%	85%	1.33	0.24
Model_41: logIL6 logMXA NGAL	0.736 [0.658 ; 0.815]	91%	30%	48%	82%	1.29	0.3
Model_38: logIL6 logIP_10 logMXA	0.706 [0.625 ; 0.787]	88%	32%	48%	79%	1.29	0.37
Model_6: NGAL	0.791 [0.720 ; 0.862]	97%	23%	48%	92%	1.26	0.13
Model_56: NGAL logIP_10 logMXA	0.752 [0.677 ; 0.828]	93%	26%	47%	83%	1.26	0.28
Model_8: logPCT NGAL	0.803 [0.731 ; 0.874]	93%	26%	47%	83%	1.24	0.29
Model_10: logPCT logIL6	0.753 [0.675 ; 0.831]	93%	25%	47%	82%	1.23	0.3
Model_14: NGAL logMXA	0.748 [0.671 ; 0.826]	93%	24%	47%	82%	1.22	0.3
Model_19: logMXA logIP_10	0.673 [0.588 ; 0.758]	87%	28%	47%	75%	1.21	0.47
Model_84: logPCT NGAL logIP_10 logIL6	0.756 [0.677 ; 0.836]	96%	19%	46%	86%	1.18	0.23
Model_18: NGAL logIP_10	0.756 [0.678 ; 0.834]	96%	15%	45%	82%	1.12	0.3
Model_24: logIP_10 CRP	0.705 [0.624 ; 0.785]	97%	14%	45%	87%	1.12	0.22
Model_5: logMXA	0.673 [0.588 ; 0.759]	87%	22%	44%	70%	1.11	0.6
Model_7: logPCT	0.771 [0.695 ; 0.847]	94%	7%	42%	64%	1.02	0.79
Model_4: logIP_10	0.575 [0.485 ; 0.664]	99%	3%	42%	75%	1.02	0.47

Models achieving the targeted performance criteria are highlighted in green (light green: LR+≥ 5·67 but < 8·5 and/or LR- ≤ 0·5 but > 0·3; dark green: LR+ ≥ 8·5 and LR- ≤ 0·3).

The best performing model in the global population (model 57) is highlighted in red.

Abbreviations: AUC, area under the curve; CI, confidence interval; LR-, negative likelihood ratio; LR+, positive likelihood ratio; NPV, negative predictive value; PPV, positive predictive value.
