# Supplementary material for: Performance Evaluation of Host Biomarker Combinations for the Diagnosis of Serious Bacterial Infection in Young Febrile Children: A Double-Blind, Multicentre, Observational Study
Source: J Clin Med. 2022 Nov 4;11(21):6563. doi: 10.3390/jcm11216563 (PMC9657860; doi:10.3390/jcm11216563)
Supplement: Supplementary file 1 [file jcm-11-06563-s001.zip › Supplementary Materials/Table S6.pdf]

Table S6. Performances of top marker combinations using logistic regression (TEST set; see also Figure 2)

Global population (7 days – 36 months; n=240)								TRAIN Cohort
**Selected Models**	**AUC [95% CI]**	**Sensitivity**	**Specificity**	**PPV**	**NPV**	**LR+**	**LR-**	AUC [95% CI]
Model_57: NGAL logTRAIL logMXA	0.831 [0.777 ; 0.886]	76%	82%	69%	87%	4.21	0.3	0.826 [0.783 ; 0.869]
Model_63: logMXA logPCT logTRAIL	0.834 [0.778 ; 0.889]	77%	77%	64%	86%	3.29	0.3	0.830 [0.787 ; 0.874]
Model_52: CRP logMXA logTRAIL	0.824 [0.767 ; 0.880]	76%	77%	63%	86%	3.28	0.32	0.842 [0.800 ; 0.883]
Model_22: logMXA logTRAIL	0.814 [0.757 ; 0.871]	77%	73%	60%	86%	2.85	0.32	0.820 [0.776 ; 0.864]
Model_27: logTRAIL CRP	0.821 [0.766 ; 0.876]	83%	65%	56%	88%	2.37	0.26	0.815 [0.774 ; 0.857]
Model_1: CRP	0.779 [0.717 ; 0.841]	82%	63%	54%	87%	2.22	0.29	0.780 [0.731 ; 0.829]
Model_11: logPCT logTRAIL	0.829 [0.774 ; 0.883]	78%	64%	54%	85%	2.17	0.34	0.792 [0.746 ; 0.837]
Model_20: logMXA CRP	0.775 [0.707 ; 0.842]	79%	59%	50%	84%	1.93	0.35	0.815 [0.767 ; 0.863]
Model_13: logPCT logMXA	0.791 [0.726 ; 0.857]	82%	57%	50%	85%	1.88	0.32	0.804 [0.755 ; 0.853]
Model_3: logTRAIL	0.815 [0.758 ; 0.871]	87%	53%	49%	88%	1.84	0.25	0.785 [0.739 ; 0.831]
Model_5: logMXA	0.709 [0.637 ; 0.782]	88%	22%	37%	77%	1.12	0.56	0.758 [0.703 ; 0.812]
Model_7: logPCT	0.789 [0.725 ; 0.853]	94%	9%	36%	74%	1.03	0.67	0.706 [0.651 ; 0.761]

≤ 3 months (7 – 91 days; n=77)							
**Selected Models**	**AUC [95% CI]**	**Sensitivity**	**Specificity**	**PPV**	**NPV**	**LR+**	**LR-**
Model_52: CRP logMXA logTRAIL	0.895 [0.771 ; 1.000]	86%	92%	71%	97%	10.46	0.16
Model_57: NGAL logTRAIL logMXA	0.883 [0.760 ; 1.000]	79%	92%	69%	95%	9.59	0.23
Model_63: logMXA logPCT logTRAIL	0.881 [0.760 ; 1.000]	79%	92%	69%	95%	9.43	0.23
Model_22: logMXA logTRAIL	0.878 [0.756 ; 1.000]	79%	90%	65%	95%	7.99	0.24
Model_27: logTRAIL CRP	0.861 [0.751 ; 0.972]	73%	86%	55%	93%	5.05	0.31
Model_1: CRP	0.766 [0.607 ; 0.925]	60%	87%	53%	90%	4.65	0.46
Model_11: logPCT logTRAIL	0.845 [0.734 ; 0.955]	67%	85%	53%	91%	4.52	0.39
Model_3: logTRAIL	0.837 [0.723 ; 0.950]	73%	77%	44%	92%	3.25	0.34
Model_20: logMXA CRP	0.885 [0.759 ; 1.000]	86%	71%	40%	96%	2.91	0.2
Model_13: logPCT logMXA	0.876 [0.764 ; 0.989]	79%	70%	38%	93%	2.62	0.31
Model_5: logMXA	0.828 [0.685 ; 0.971]	93%	21%	21%	93%	1.18	0.34
Model_7: logPCT	0.792 [0.644 ; 0.941]	93%	12%	21%	88%	1.05	0.58

> 3 months (92 days – 36 months; n=163)							
**Selected Models**	**AUC [95% CI]**	**Sensitivity**	**Specificity**	**PPV**	**NPV**	**LR+**	**LR-**
Model_57: NGAL logTRAIL logMXA	0.782 [0.710 ; 0.854]	75%	76%	69%	81%	3.1	0.33
Model_63: logMXA logPCT logTRAIL	0.791 [0.719 ; 0.862]	77%	67%	63%	80%	2.32	0.35
Model_52: CRP logMXA logTRAIL	0.766 [0.692 ; 0.841]	74%	67%	62%	78%	2.25	0.39
Model_22: logMXA logTRAIL	0.759 [0.684 ; 0.835]	77%	62%	59%	79%	2.02	0.38
Model_27: logTRAIL CRP	0.779 [0.706 ; 0.851]	85%	52%	56%	83%	1.76	0.29
Model_1: CRP	0.738 [0.660 ; 0.815]	87%	47%	54%	83%	1.65	0.28
Model_11: logPCT logTRAIL	0.804 [0.735 ; 0.874]	81%	50%	54%	78%	1.62	0.38
Model_20: logMXA CRP	0.722 [0.640 ; 0.805]	78%	52%	54%	77%	1.61	0.43
Model_13: logPCT logMXA	0.756 [0.677 ; 0.836]	82%	48%	53%	79%	1.58	0.37
Model_3: logTRAIL	0.782 [0.709 ; 0.855]	90%	37%	50%	83%	1.42	0.28
Model_5: logMXA	0.673 [0.588 ; 0.759]	87%	22%	44%	70%	1.11	0.6
Model_7: logPCT	0.771 [0.695 ; 0.847]	94%	7%	42%	64%	1.02	0.79

Models achieving the targeted performance criteria are highlighted in green (light green: LR+≥ 5·67 but < 8·5 and/or LR- ≤ 0·5 but > 0·3; dark green: LR+ ≥ 8·5 and LR- ≤ 0·3).

The best performing model in the global population (model 57) is highlighted in red. See respective ROC curves and Forest Plots in Figure 2.

Abbreviations: AUC, area under the curve; CI, confidence interval; LR-, negative likelihood ratio; LR+, positive likelihood ratio; NPV, negative predictive value; PPV, positive predictive value.
