# Supplementary material for: Performance Evaluation of Host Biomarker Combinations for the Diagnosis of Serious Bacterial Infection in Young Febrile Children: A Double-Blind, Multicentre, Observational Study
Source: J Clin Med. 2022 Nov 4;11(21):6563. doi: 10.3390/jcm11216563 (PMC9657860; doi:10.3390/jcm11216563)
Supplement: Supplementary file 1 [file jcm-11-06563-s001.zip › Supplementary Materials/Table S7.pdf]

Table S7. Performances of marker and clinical variable combinations using machine learning (TEST set)

Global population (7 days – 36 months; n=240)								TRAIN Cohort
**Models**	**AUC [95% CI]**	**Sensitivity**	**Specificity**	**PPV**	**NPV**	**LR+**	**LR-**	AUC [95% CI]
**gbm_5CV + TRAIL + MxA**	0.826[0.770;0.880]	74%	80%	66%	85%	3.621	0.329	0.854[0.815;0.893]
gamLoess_5CV + PCT + MxA	0.819[0.762;0.874]	67%	80%	64%	82%	3.376	0.415	0.849[0.806;0.891]
glmboost_5CV + PCT + TRAIL	0.827[0.772;0.882]	74%	78%	64%	85%	3.311	0.337	0.833[0.792;0.873]
gam_5CV + CRP + MxA	0.807[0.747;0.866]	74%	75%	61%	84%	2.897	0.351	0.851[0.809;0.893]
gbm_5CV + TRAIL	0.819[0.762;0.875]	80%	72%	60%	87%	2.846	0.281	0.849[0.809;0.889]
glmboost_5CV + CRP + TRAIL	0.818[0.761;0.874]	75%	73%	60%	85%	2.804	0.341	0.844[0.804;0.884]
gamLoess_CRP + TRAIL	0.820[0.765;0.875]	80%	67%	56%	86%	2.408	0.303	0.825[0.784;0.866]
gamLoess_PCT + TRAIL	0.831[0.776;0.884]	85%	62%	55%	88%	2.249	0.248	0.800[0.755;0.845]
gamLoess_CRP + MxA	0.797[0.735;0.859]	76%	65%	54%	84%	2.175	0.366	0.840[0.797;0.884]
ctree_5CV + CRP	0.791[0.732;0.850]	82%	62%	54%	87%	2.149	0.289	0.818[0.777;0.860]
gamLoess_5CV + PCT	0.775[0.711;0.838]	85%	55%	50%	87%	1.869	0.283	0.792[0.744;0.841]
gamLoess_5CV + MxA	0.773[0.710;0.834]	87%	52%	49%	88%	1.819	0.251	0.807[0.759;0.855]
glm_TRAIL	0.813[0.756;0.869]	87%	52%	49%	88%	1.795	0.254	0.785[0.739;0.832]
pls_PCT + MxA	0.798[0.732;0.862]	83%	51%	48%	85%	1.699	0.327	0.805[0.757;0.852]
glm_MxA	0.711[0.639;0.782]	87%	28%	39%	80%	1.207	0.467	0.761[0.708;0.815]
glm_CRP	0.773[0.709;0.835]	95%	18%	38%	88%	1.159	0.267	0.783[0.735;0.832]
slda_5CV	0.641[0.567;0.714]	96%	8%	36%	81%	1.051	0.431	0.691[0.635;0.746]
glm_PCT	0.789[0.725;0.851]	95%	7%	35%	73%	1.024	0.68	0.714[0.659;0.768]

≤ 3 months (7 – 91 days; n=77)							
**Models**	**AUC [95% CI]**	**Sensitivity**	**Specificity**	**PPV**	**NPV**	**LR+**	**LR-**
multinom_5CV + MxA + CRP	0.866[0.754;0.977]	50%	98%	89%	88%	31.000	0.508
gam_5CV + MxA + PCT	0.919[0.836;1.000]	69%	97%	85%	92%	21.313	0.323
knn_PCT + MxA	0.903[0.828;0.978]	69%	97%	85%	92%	21.313	0.323
knn_CRP + MxA	0.903[0.816;0.989]	69%	95%	79%	92%	14.208	0.328
**slda_5CV + MxA + TRAIL**	0.893[0.800;0.986]	75%	92%	71%	93%	9.300	0.272
ctree_5CV + TRAIL	0.857[0.752;0.962]	69%	89%	61%	92%	6.089	0.352
ctree_5CV + CRP + TRAIL	0.846[0.725;0.967]	69%	89%	61%	92%	6.089	0.352
knn_CRP + TRAIL	0.850[0.743;0.956]	69%	87%	58%	92%	5.328	0.359
gam_5CV + PCT + TRAIL	0.865[0.775;0.864]	56%	87%	53%	89%	4.359	0.502
pls_PCT + TRAIL	0.848[0.743;0.951]	56%	86%	50%	88%	3.875	0.512
glm_TRAIL	0.840[0.731;0.947]	75%	81%	50%	93%	3.875	0.31
glm_CRP	0.743[0.586;0.899]	50%	87%	50%	87%	3.875	0.574
lda_5CV + CRP	0.760[0.609;0.910]	69%	79%	46%	91%	3.279	0.395
glm_MxA	0.824[0.691;0.955]	75%	74%	43%	92%	2.906	0.337
dwdLinear_5CV + PCT	0.811[0.690;0.932]	56%	81%	43%	88%	2.906	0.543
glm_PCT	0.793[0.654;0.931]	88%	69%	42%	96%	2.855	0.18
glm_5CV + MxA	0.855[0.752;0.957]	81%	66%	38%	93%	2.399	0.284
knn_5CV	0.622[0.478;0.765]	100%	13%	23%	100%	1.148	0

> 3 months (92 days – 36 months; n=163)							
**Models**	**AUC [95% CI]**	**Sensitivity**	**Specificity**	**PPV**	**NPV**	**LR+**	**LR-**
gamLoess_5CV + CRP + TRAIL	0.800[0.731;0.869]	69%	75%	66%	77%	2.736	0.413
glmboost_5CV + PCT + TRAIL	0.793[0.722;0.864]	78%	67%	63%	81%	2.389	0.327
knn_CRP + TRAIL	0.792[0.721;0.863]	85%	60%	60%	85%	2.132	0.245
knn_PCT + MxA	0.753[0.679;0.827]	77%	63%	60%	79%	2.076	0.373
**gbm_5CV + MxA + TRAIL**	0.782[0.711;0.853]	82%	60%	60%	83%	2.059	0.294
gamLoess_5CV + MxA + CRP	0.754[0.678;0.829]	82%	60%	60%	83%	2.059	0.294
gbm_5CV + PCT	0.752[0.675;0.828]	56%	73%	59%	70%	2.042	0.607
gbm_5CV + TRAIL	0.798[0.727;0.868]	85%	58%	59%	85%	2.026	0.254
slda_5CV + MxA + PCT	0.770[0.696;0.842]	78%	59%	58%	79%	1.899	0.374
gbm_5CV + MxA	0.710[0.629;0.790]	81%	57%	57%	81%	1.874	0.336
gamLoess_5CV + CRP	0.757[0.682;0.831]	75%	58%	56%	76%	1.781	0.432
gamLoess_PCT + TRAIL	0.809[0.740;0.877]	88%	48%	55%	85%	1.711	0.243
gbm_5CV	0.663[0.576;0.750]	68%	58%	54%	71%	1.607	0.559
glm_CRP	0.738[0.660;0.814]	90%	39%	51%	84%	1.469	0.264
glm_TRAIL	0.782[0.708;0.855]	96%	25%	48%	89%	1.279	0.175
knn_CRP + MxA	0.740[0.660;0.819]	93%	19%	45%	78%	1.143	0.388
glm_MxA	0.673[0.587;0.759]	94%	13%	44%	75%	1.077	0.466
glm_PCT	0.774[0.698;0.848]	96%	7%	43%	70%	1.032	0.599

Models achieving the targeted performance criteria are highlighted in green (light green: LR+ ≥ 5·67 but < 8·5 and/or LR- ≤ 0·5 but > 0·3; dark green: LR+ ≥ 8·5 and LR- ≤ 0·3).

The best performing model in the global population is in bold.

Abbreviations: AUC, area under the curve; CI, confidence interval; LR-, negative likelihood ratio; LR+, positive likelihood ratio; NPV, negative predictive value; PPV, positive predictive value.
